# Multidirectional Characterization of Phytochemical Profile and Health-Promoting Effects of *Ziziphora bungeana* Juz. Extracts

**DOI:** 10.3390/molecules27248994

**Published:** 2022-12-16

**Authors:** Karlygash Zhaparkulova, Aigerim Karaubayeva, Zuriyadda Sakipova, Anna Biernasiuk, Katarzyna Gaweł-Bęben, Tomasz Laskowski, Aliya Kusniyeva, Azamat Omargali, Tolkyn Bekezhanova, Liliya Ibragimova, Galiya Ibadullayeva, Amangeldy Jakiyanov, Karolina Czech, Kuanysh Tastambek, Kazimierz Głowniak, Anna Malm, Wirginia Kukula-Koch

**Affiliations:** 1 School of Pharmacy, S.D. Asfendiyarov Kazakh National Medical University, Tole-bi 94, Almaty 050012, Kazakhstan; 2 Chair and Department of Pharmaceutical Microbiology, Faculty of Pharmacy, Medical University of Lublin, 1, Chodzki str., 20-093 Lublin, Poland; 3 Department of Cosmetology, University of Information Technology and Management in Rzeszów, 2 Sucharskiego str., 35-225 Rzeszów, Poland; 4 Department of Pharmaceutical Technology and Biochemistry and BioTechMed Centre, Faculty of Chemistry, Gdańsk University of Technology, Gabriela Narutowicza Str. 11/12, 80-233 Gdańsk, Poland; 5 Edinburgh Dental Institute, The University of Edinburgh, Edinburgh EH8 9YL, UK; 6 Department of Fundamental Medicine, Al-Farabi Kazakh National University, Almaty 050040, Kazakhstan; 7 Department of Chemical and Biochemical Engineering, Geology and Oil-Gas Business Institute Named after K. Turyssov, Institute of Chemical and Biological Technologies, Satbayev University, Almaty 050043, Kazakhstan; 8 Ecology Research Institute, Khoja Akhmet Yassawi International Kazakh-Turkish University, Turkistan 161200, Kazakhstan; 9 Department of Pharmacognosy, Medical University of Lublin, 1, Chodźki str., 20-093 Lublin, Poland

**Keywords:** *Ziziphora*, antimicrobial activity, tyrosinase inhibition, antioxidant potential, flavonoids, HPLC-MS, erythrocyte lysis, Lamiaceae, cytotoxicity

## Abstract

*Ziziphora* species (Lamiaceae) have been used in traditional medicine as sedatives, antiseptics, carminatives, or expectorants. Despite their common applications in phytotherapy, there is still lack of evidence about the composition of their extracts and its impact on biological properties of the plants. The aim of this study was to evaluate the content of *Ziziphora bungeana*, a less studied species growing in Kazakhstan, using HPLC-ESI-QTOF-MS/MS instrumentation and to determine its antimicrobial, antioxidant, and cytotoxic activity together with inhibitory properties against tyrosinase and toxicity in erythrocyte lysis assay. Extracts from *Z. bungeana* were found to be sources of flavonoids, phenolic acids, organic acids, and terpenes that determined their antiradical activity. The minimum inhibitory concentrations of extracts were lower for Gram-positive bacteria (1.25–10 mg/mL) than for Gram-negative bacteria and fungi (5–20 mg/mL). The EC_50_ value calculated for antiradical activity ranged between 15.00 ± 1.06 µg/mL and 13.21 ± 3.24 µg/mL for ABTS and DPPH assays, respectively. *Z. bungeana* extracts were found to decrease the activity of tyrosinase by 50% (at 200 µg/mL) similarly to kojic acid and were slightly cytotoxic for human melanoma A375 cell line (at 200 µg/mL) with no effect on HaCaT keratinocytes. In the end, *Z. bungeana* did not reveal toxic effects in hemolytic assay as compared to the positive control Triton X-100. The performed tests show potential application of the plant in the treatment of infectious diseases, disorders caused by free radicals, and skin problems.

## 1. Introduction

Plant biodiversity is certainly an invaluable wealth of our planet. Herbal remedies have been used in the treatment of various diseases for centuries as plants are capable of synthesizing a wide range of metabolites of pharmacological significance. Traditionally plants were used as whole, in the form of the total extracts to enable the combined action of their ingredients. From a therapeutic point of view, the natural products, organic low-molecular structures called secondary metabolites, are beneficial to both the plants, as they protect them from harsh environmental factors or pathogens, and to humans for their medicinal or cosmetic applications. A multitude of compounds that are encountered in the world’s flora inspires the scientists to modify the original scaffoldings and to create more active, stable, or less toxic molecules to fight the diseases that are difficult to treat with conventional drugs. This work presents a compositional study and biological activity determinations that are performed on the *Ziziphora bungeana* species collected in Kazakhstan. This species will be tested for its composition and activity, the results of which will allow for determining of antimicrobial properties and potential application of the plant as an additive to drugs or foods, an ingredient of cosmetic products with anti-aging, anti-cancer, or whitening properties for the use in skin cancer therapy, and whether these extracts are safe. The undertaken direction of studies was inspired by former research data that proved a diversified composition of *Ziziphora* spp. and a marked biological potential of its extracts.

The genus *Ziziphora* L. (Lamiaceae) comprises about 30 species widespread all over Asia, Africa, and Europe that represent the prototypical example of the Lamiaceae family. Plants from this genus are known to produce monoterpenes, triterpenes and phenolic substances belonging mainly to the group of flavonoids [1,2,3]. Essential oil (EO) from *Ziziphora clinopodioides* and *Z. tenuior* was proven to be rich in pulegone, menthone, and limonene, whereas more polar extracts were characterized by a high phenolic content [4,5]. Rich composition in secondary metabolites allows for a multi-directional application of the plants. They are used in the form of infusions, decoctions and macerates as remedies for stomachache, common cold, inflammatory conditions, cough, migraine, fever, depression, diarrhea, and gastrointestinal diseases. Moreover, they are known from their sedative, expectorant, antiseptic, and carminative properties [2,4,6,7,8,9]. In Kazakh traditional medicine, *Ziziphora* species possess several medicinal uses. In particular *Z. bungeana* and *Z. clinopodioides* are administered in the treatment of cardiovascular system disorders or infections [10].

Despite the presence of polyphenols in the extracts that have impact on the antioxidant potential of the mentioned plant species, the constituents of less polar fractions may also influence the total antiradical potential of the plant, affect its antispasmodic, anti-inflammatory, anti-infective, and expectorant properties, which broadens their activity range [11].

Having in mind plentiful applications of different *Ziziphora* species, the aim of the study was to deliver information about another less known *Ziziphora bungeana* Juz., which is a synonym of *Z. clinopodioides* ssp. *bungeana*. This plant is distributed mainly in Kazakhstan, China, Central Asia, and Mongolia [12]. The scientific literature is still lacking sufficient information about its composition and beneficial actions, including the antimicrobial, antifungal, antioxidant, or skin whitening potential, whose determination will be performed in this study together with compositional analysis by HLPC-ESI-QTOF-MS/MS technique. Based on the scientific literature data on *Ziziphora* gender, this plant is expected to be a good natural antioxidant and antibacterial agent with high potential for its application in foods and cosmetics as a preservative and antiradical component. This study was designed to meet the diverse expectations towards plant extracts in the context of their potential use in the treatment of civilization diseases or in the skin care because of the harmful effects caused by the environment. The determination of the antioxidant, antimicrobial, and whitening properties of the extracts of different polarities will allow for the study on the potential use of *Ziziphora bungeana* in cosmetics. Moreover, the toxicity studies in relation to normal and cancer skin cells will bring evidence for a discussion about safety of its use. On the other hand, the qualitative analysis of different polarity extracts will develop a fingerprint responsible for the determined action of the plant.

## 2. Results and Discussion

### 2.1. Chemical Composition of Extracts by HPLC-ESI-QTOF-MS/MS

The applied chromatographic method was capable of separating metabolites present in the extracts from the aerial parts of *Z. bungeana*, whereas the applied mass spectrometer settings provided MS/MS spectra that helped in the identification of metabolites from different groups.

**Figure 1:**
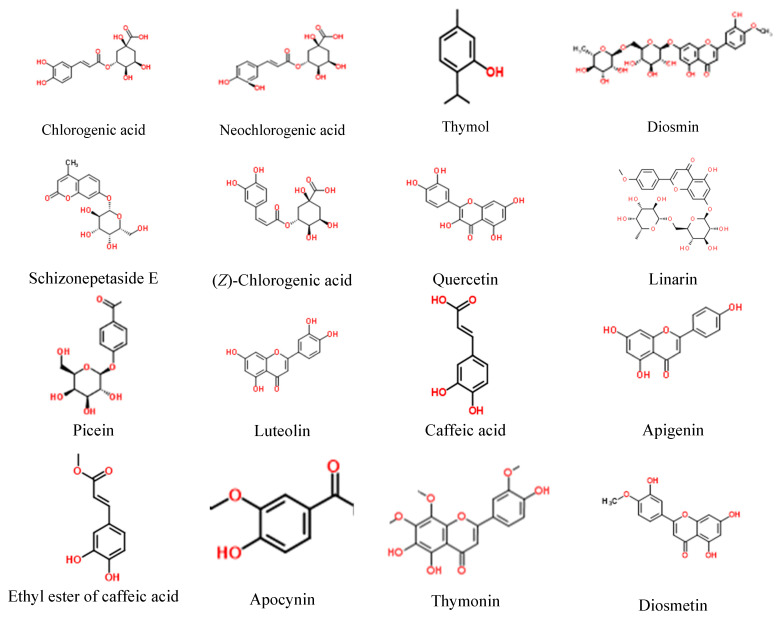

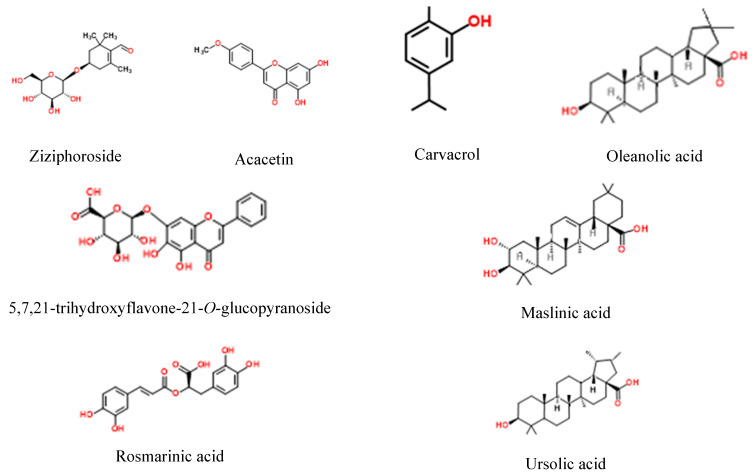
Structure of the components identified in *Ziziphora bungeana* extracts.

The HPLC-ESI-QTOF-MS/MS analysis confirmed the presence of phenolic acids, flavonoids, triterpenes, and monoterpene glucosides in the tested extracts. The list of 26 tentatively identified components is presented in the Table 1 and Figure 1, whereas the recorded mass chromatograms from positive and negative ionization modes are shown in the Supplementary File (Figures S1 and S2). It was proven that the components of *Z. bungeana* were previously described in other species from the same gender.

The applied LC-MS technology enabled the identification of components, e.g., thymol, carvacrol, or ziziphorosides, that were previously determined in *Ziziphora* spp. by GC-MS technique. Milder fragmentation conditions (fragmentation voltage of 110 V, capillary voltage of 3000 V, gas temperature of 275 °C and collision energy of 10 V) increased the chance to observe terpene compounds in the chromatogram in the liquid chromatography-based system. Ziziphoroside isomers, possibly ziziphoroside A, B, and C, were present in the mass spectra in the form of adducts with sodium ions. The remaining compounds were traced in the form of molecular ions with or without a proton.

*Z. bungeana* was proven to contain different types of metabolites whose presence was revealed in the HPLC-MS assessment of the extracts tested in this study. Among them flavonoids constituted the leading group of components, followed by phenolic acids and, interesting from the structural point of view, terpenes, e.g., ziziphorosides or schizonepetaside E. It is worthwhile to note that the scientific literature still lacks sufficient information about the composition of this plant species. For the moment, to the best of the authors’ knowledge, there is only one original manuscript that discusses the composition of extracts based on the HPLC-MS results. The researchers confirmed the presence of twelve flavonoids in *Z. bungeana*, that included: kaempferol-7-*O*-rutinoside, kaempferol-3-*O*-rutinoside; rutin; apigenin-7-*O*-rutinoside; 3′-hydroxyacacetin-7-*O*-rutinoside; acacetin-4′-*O*-rutinoside; pinocembrin-7-*O*-rutinoside; chrysin-7-*O*-rutinoside; linarin; 5,7,3′-trihydroxy-6,4′,5′-trimethoxyflavone, 5,4′-dihydroxy-6-methoxy-7,8-methylenedioxyflavone, and 5,7-dihydroxy-6-methoxyflavone. The above list of components presented in the Table 1 expands information on the composition of this species.

The analysis of previously published papers provided a more detailed list of components of *Ziziphora* genus that helped to enrich the list of the tentatively identified metabolites of *Z. bungeana* extracts. Previous investigation of the chemical profile of ethyl acetate, methanol, and water extracts from the aerial parts of *Ziziphora taurica* subsp. *cleonioides* showed that among the identified compounds, rosmarinic acid and chlorogenic acid were the most abundant components of the methanol extract with the calculated concentration of 3375.67 ± 38.02 and 3225.10 ± 16.44 µg/mL, respectively [32]. Both compounds were also determined in the studied species. Moreover, flavonoids constituted the major group of bioactive compounds present in *Ziziphora clinopodioides* Lam. [2] together with organic acids, alkaloids, and glycosides that were listed by other authors [33].

As mentioned above, *Ziziphora* species belong to the plants that synthesize secondary metabolites from different classes, which explains their various therapeutical applications. In the previous studies *Ziziphora* spp. were found to be rich sources of phenolic compounds. Interestingly, both extracts and the EO were sources of polyphenols. For example, the measured total phenolic content in *Z. tenuior* was equal to 49.0 ± 1.4 mg mg gallic acid/100 g of EO [15].

The metabolites that are mentioned above are important from the pharmacological point of view. Phenolic acids and flavonoids are known scavengers of free radicals that are efficiently inhibiting the progression of different inflammatory conditions and civilization diseases progressing with an important role of radicals [34]. Their presence in the final extracts from edible plants is certainly related to the type of the plant, but also to the extraction conditions [35].

Based on this information, the authors found it crucial to study the biological potential of *Z. bungeana* and focus on its antimicrobial, antioxidant, and anti-tyrosynase properties, as well as to evaluate safety.

### 2.2. Antimicrobial Activity Assessment

The data presented in Table 2 and Table 3 indicate that the extracts from *Ziziphora bungeana* showed some potential antimicrobial activity. They were more effective against reference Gram-positive bacteria than towards Gram-negative bacteria and yeasts. The lowest concentration of extracts which inhibited the growth of these microorganisms or killed them ranged from 1.25 mg/mL to 20 mg/mL and from 2.5 mg/mL to 20 mg/mL, respectively.

As shown in Table 2, in the case of Gram-positive bacteria, the MIC values of the extracts were in the range of 1.25–10 mg/mL. Their activity was the same towards staphylococci, both *Staphylococcus aureus* ATCC 43,300 (MRSA—Methicillin Resistant *S. aureus*), *S. aureus* ATCC 29,213 (MSSA—Methicillin Susceptible *S. aureus*) and *Staphylococcus epidermidis* ATCC 12,228 with MIC = 2.5 mg/mL and MBC = 2.5–5 mg/mL (except *S. aureus* ATCC 29213; MIC = 5 mg/mL for Z2). In turn, *Micrococcus luteus* ATCC 10,240 was the most (MIC = 1.25 mg/mL and MBC = 5 mg/mL) and the least sensitive (MIC = 5 mg/mL and MBC = 10 mg/mL) to Z2 and Z3, respectively. The antibacterial effect against two reference *Bacillus* spp. strains was lower. MIC values were mainly 5 mg/mL. However, MIC = 2.5 mg/mL was shown for the Z3 against *B. subtillis* ATCC 6633 and MIC = 10 mg/mL for Z1 towards *B. cereus* ATCC 10876. MBCs of all extracts for bacilli were the same—10 mg/mL.

The activity of extracts towards Gram-negative rods-shaped, was slightly weaker with MIC = 5–20 mg/mL and MBC = 10–20 mg/mL. Among them, Z2 showed the highest effect towards *Bordetella bronchiseptica* ATCC 4617 (MIC = 5 mg/mL and MBC = 20 mg/mL). For Z1, MIC = 10 mg/mL against *B. bronchiseptica* and *Pseudomonas aeruginosa* ATCC 9027 was shown. In the case of Z2, the same MIC value towards reference *Klebsiella pneumoniae* strain was indicated. The growth of remaining Gram-negative bacteria was inhibited by these extracts at a concentration of 20 mg/mL.

**Figure 2:**
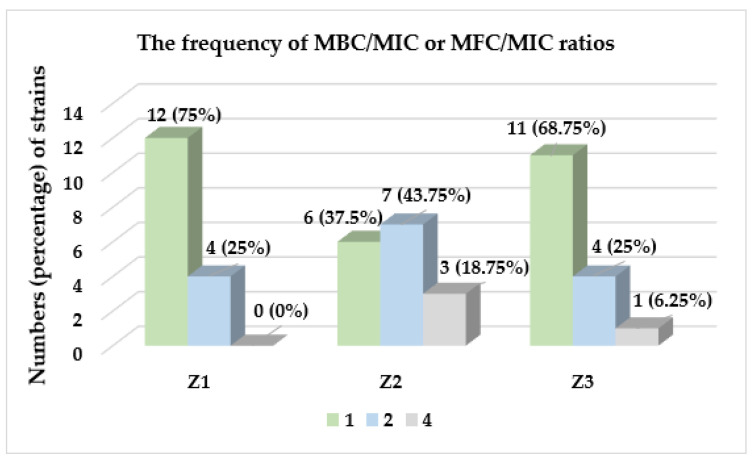
The frequency of occurrence of particular MBC/MIC or MFC/MIC ratios of *Z. bungeana* extracts against the reference strains of bacteria and fungi used in the study.

Taking into account the MBC/MIC and MFC/MIC ratios, as presented at Figure 2, it was shown that extracts from *Z. bungeana* had a beneficial bactericidal or fungicidal effect towards reference microorganisms. The values of MBC/MIC or MFC/MIC were in the range 1–4. MICs equal to MBC or MFC (MBC/MIC = 1 and MFC/MIC = 1) were shown for most, i.e., 12 (75%) and 11 (68.75%) strains in the case of Z1 and Z3, respectively. For Z2, these values were different. However, these ratios were mainly 1 (for six (37.5%) strains) and 2 (for seven (43.75%) strains). The value of 4 was found the least frequently (only for Z2 and Z3). The bacteriostatic effect (MBC/MIC > 4 or MFC/MIC > 4) of the tested extracts was not demonstrated.

In the next stage of this study, the total antimicrobial activity (TAA) was assessed. The total antibacterial activity or total antifungal activity of the studied extracts Z1–Z2 was shown in Table 4. The MIC and TAA, both total antibacterial activity and total antifungal activity values are important pharmacological tools. They are useful in determining the activity of extracts in mg/mL (potency) of plants extracts for isolating bioactive compounds and total activity on mL/g (efficacy) for the selection of appropriate plant species [36]. Generally, their TAA values were the highest against Gram-positive bacteria: *Staphylococcus* spp., *Micrococcus luteus*, followed by *Bacillus* spp. (3.35 ± 0.0–20.20 ± 0.0 mL/g) and the lowest against Gram-negative bacteria and fungi belonging to *Candida* spp. (1.09 ± 0.0–4.21 ± 1.46 mL/g). Z1 had higher TAA towards reference strains of *S. aureus, S. epidermidis* and *M. luteus* (13.39 ± 0.0 mL/g). TAA values of Z2 varied slightly and were in the range 4.21 ± 1.46 to 20.20 ± 0.0 mL/g against these bacteria. In turn, TAA values of Z3 were slightly lower (4.38 ± 0.0–8.75 ± 0.0 mL/g).

**Figure 3:**
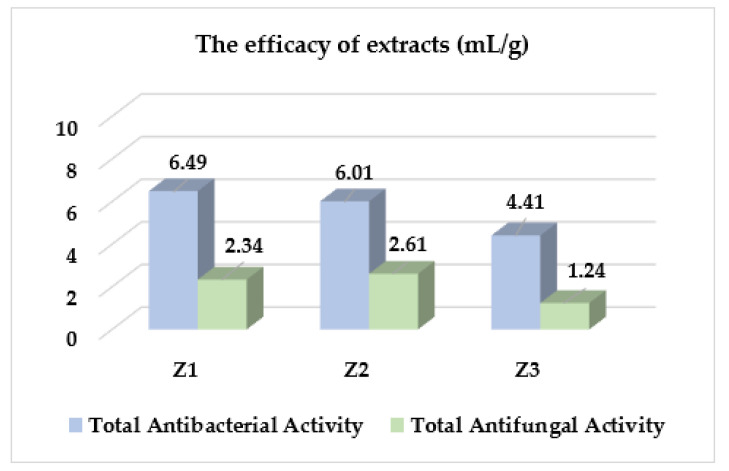
The efficacy (mean TAA values, mL/g) of *Z. bungeana* extracts against all the studied reference bacteria and fungi.

As shown in Figure 3, Z1, Z2, and Z3 extracts had the mean total antibacterial activities of 6.49, 6.01 and 4.41 mL/g, respectively. In turn, the mean total antifungal activity of extracts was lower in the range 1.24–2. mL/g. In conclusion, Z1 and Z2 had a similar and better efficacy against both bacteria and fungi compared to Z3. The higher the TAA value, the more efficacious the plant extract.

The results indicated that Z1–Z3 extracts from *Z. bungeana* showed some antimicrobial activity with bactericidal or fungicidal effect. Among all studied reference microorganisms, Gram-positive bacteria were the most sensitive to them. The lowest concentrations of Z1–Z3 extracts which inhibited the growth of the tested microorganisms or killed them ranged from 1.25 mg/mL to 20 mg/mL and from 2.5 mg/mL to 20 mg/mL, respectively. Overall, the Gram-positive bacteria were more sensitive to the extracts than the Gram-negative bacteria and yeasts from *Candida*. The difference in the sensitivity between these microorganisms may be due to the variation in their cell wall structure. The Gram-positive bacterial cell wall consists of 70–100 layers of peptidoglycans. Peptidoglycan is comprised of two polysaccharides, *N*-acetyl-glucosamine and *N*-acetyl-muramic acid cross-linked by peptide side chains and cross bridges [37]. It is possible that active compounds from extracts can easier break important bonds in cell wall structure in these bacteria. On the other hand, the cell wall of Gram-negative bacteria is far more complex, and it is among other things the reason they are more resistant for biologically active compounds.

There is little information in the literature on the biological activity of *Z. bungeana* extracts. However, there are reports on other *Ziziphora* species. Some authors showed antimicrobial effect of different extracts, EO or selected compounds derived from *Ziziphora* gender. The results and findings described herein are in accordance with some other studies.

The antibacterial activity of EO and its two main components (pulegone and 1,8-cineole) obtained from the aerial flowering parts of *Ziziphora clinopodioides* subsp. *bungeana* (Juz.) Rech. f. was analyzed by Sonboli A. et al. [38] against seven bacteria. It was found that the EO exhibited interesting activity against *S. epidermidis, S. aureus, E. coli,* and *B. subtilis* with MIC values of 3.75 mg/mL. These results were similar to ours for Gram-positive bacteria.

In turn, the inhibitory effect of methanol extract and EO from *Ziziphora persica* was tested against 98 strains belonging to 51 bacteria species by standard dilution methods. The results showed that both extract and EO had antibacterial activity against many tested bacteria. The lowest MIC values (7.81 µg/mL) of EO were obtained against *Bacillus dipsauri, Corynebacterium cystitidis*, and *Corynebacterium flavescens* [39]. 

*Z. clinopodioides* was studied by subsequent researchers. The LC-MS/MS results of Özkan E.E. et al. [3] indicated that quinic acid, malic acid and rhoifolin are the abundant compounds in aerial and root ethanol extracts of *Z. clinopodioides*. Both extracts exhibited moderate antifungal activity with MIC = 39.06 μg/mL against *Candida tropicalis.* Moreover, these extracts showed some better or the same antibacterial effect against reference *S. aureus, S. epidermidis*, and *E. faecalis* strains (MIC = 0.312–1.25 mg/mL) as our extracts of *Z. bungeana*. In the case of Gram-negative bacteria (*P. aeruginosa, E. coli, K. pneumoniae* and *P. mirabilis*) and *C. albicans*, no activity was showed.

Moreover, the studies of Anzabi Y. et al. [40] showed that the *Z. clinopodioides* EOs was effective on many tested bacteria and can be used as natural antimicrobial drug against microorganisms causing urogenital tract infections in women. The aerial parts of *Z. clinopodioides* were also screened by other authors [41] for their possible antimicrobial activities. Methanol extract was found to have moderate antimicrobial activity against some microorganisms tested. *Acinetobacter lwoffii* and *Candida krusei* were the most sensitive for this extract. The antimicrobial properties were also found in *Z. clinopodioides* EOs collected from provinces in western Iran. The studied EO inhibited the growth of *Listeria monocytogenes, S. typhimurium, E. coli* O157:H7, *B. subtilis, B. cereus*, and *S. aureus* at MIC values between 0.03% and 0.04%. The Gram-positive bacteria were the most susceptible to it, while Gram-negative bacteria were resistant [1]. The interesting antibacterial activity against seven Gram-positive or Gram-negative bacteria exhibited also EO and methanol extract of *Z. clinopodioides* subsp. *rigida* (BOISS.) RECH. f. from Iran. The obtained results indicated that *B. subtilis* was the most sensitive microorganism to this EO, with the lowest MIC = 3.8 mg/mL. The growth inhibition of *S. epidermidis* and *S. aureus* was observed at similar MIC = 7.5 mg/mL. The inhibitory activity of EO against *E. faecalis, K. pneumoniae*, and *E. coli* was also determined with MIC values equal to or greater than 15 mg/mL. No activity was observed against *P. aeruginosa* [42].

The subsequent results of Hazrati et al. [2] showed 17 and 21 different compounds (comprising 99.7% of total EO) in *Z. clinopodioides* and *Z. tenuior*, respectively. The major identified compounds in EO analysis reported as pulegone and menthone for *Z. clinopodioides*, or pulegone and limonene for *Z. tenuior*. Both *Ziziphora* species were also rich in phenolic compounds. These authors investigated the antibacterial activity of EOs against important foodborne pathogenic bacteria and showed that they could be considered as good sources of natural antibacterial material as well as food preservative [2,15]. 

Additionally, Celiket al. [43] evaluated the antimicrobial and anti-biofilm properties of *Z. tenuior*. EO against multi-drug resistant *Acinetobacter baumannii* with MIC = 0.6–1.25 μL/mL and MBC = 2.5–5.0 μL/mL. Furthermore, minimal biofilm inhibition concentration (MBIC) values of 0.3–1.25 μL/mL and minimal biofilm eradication concentration (MBEC) values of 5–10 μL/mL were observed.

Considering all above information, it can be concluded that *Ziziphora* plants may deliver extracts that are important from a pharmacological point of view, as they may help combat the occurring bacterial and fungal infections.

### 2.3. Antioxidant Activity Assessment

**Figure 4:**
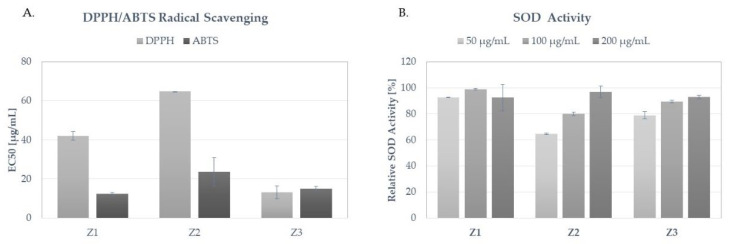
Antioxidant activity of Z1, Z2 and Z3 extracts from *Z. bungeana*: (**A**) neutralization of DPPH and ABTS free radicals, displayed as EC_50_; (**B**) relative activity of superoxide dismutase (SOD) of Z1, Z2 and Z3 extracts; histograms show mean values ±SD, *n* = 3.

Antioxidant activity of *Ziziphora bungeana* extracts was compared using DPPH and ABTS radical scavenging assays (Figure 4A) and determination of superoxide dismutase (SOD) activity (Figure 4B). In respect of the radical scavenging potential, extract Z3 showed the most significant activity with EC_50_ values of 15.00 ± 1.06 µg/mL and 13.21 ± 3.24 µg/mL for ABTS and DPPH assays, respectively. All tested extracts also showed significant SOD activity, dependent on the extract concentration. The most effective was extract Z1, showing > 90% SOD activity in all three tested concentrations. Extract Z2 was the least effective. At the concentration of 50 µg/mL, the mean SOD activity detected for this extract was 64.4 ± 0.55%.

The antioxidant activity of 50% (*v*/*v*) ethanolic extract from *Z. bungeana* was recently compared with other plants from Lamiaceae family by measuring its influence on the level of lipid peroxidation in the liver microsome and the membrane-stabilizing properties [44]. The antioxidant potential of the extracts was significant in both assays but moderate in comparison with other Lamiaceae plants. 

However in the publication of Gursoy and co-investigators [41], the aerial parts of *Z. clinopodioides* were found to be the strongest radical scavengers among the tested species from Lamiaceae family, namely: *Z. clinopodioides, Cyclotrichium niveum*, and *Mentha longifolia* subsp. *typhoides* var. *typhoides* in the DPPH and beta-carotene/linoleic acid assays. The calculated IC_50_ values for the tested extracts were 37.73 ± 1.18 µg/mg for DPPH and 83.56 ± 1.19% in the inhibition capacity of the linoleic acid. Moreover, the total phenolic content of its methanolic extract was the highest among the tested species and was equal to 129.55 +/− 2.26 µg/mg.

Recently, the antioxidant activity of the aqueous, ethyl acetate, and methanolic extracts from other *Ziziphora* species, *Ziziphora taurica* subsp. *taurica*, were compared using DPPH and ABTS scavenging assays. In both assays, the methanolic ectract from *Z. taurica* was the most effective with IC_50_ values of 5.74 ± 0.08 mg/mL and 2.74 ± 0.10 mg/mL for DPPH and ABTS scavenging, respectively. The EC_50_ values obtained in our study suggest that the antioxidant potential of *Z. bungeana* extracts is higher than that of *Z. taurica* [45].

### 2.4. Tyrosinase Activity Assay

Tyrosinase (EC 1.14.18.1) is a cooper containing metalloenzyme catalyzing the first two, rate-limiting steps of mammalian melanogenesis. Neither increased nor decreased activation of tyrosinase is desirable as it may lead to hyper- or hypopigmentation disorders, respectively. Natural extracts and compounds with tyrosinase inhibitory activity are particularly desired by the cosmetic industry as they serve as active ingredients in skin lightening cosmetics and rituals [46]. On the other hand, the compounds increasing the activity of tyrosinase might be considered as topical treatment for vitiligo [47]. 

Investigating the influence of novel extracts and compounds on tyrosinase activity is commonly performed using the assay utilizing commercially available mushroom tyrosinase, incubated with its substrate L-3,4-dihydroxyphenylalanine (L-DOPA), in the presence or absence of tested compound. Despite the low costs, simplicity, and high throughput of the procedure, the assay has several limitations, resulting from substantial differences between mushroom and mammalian tyrosinase [48]. Therefore, the influence of plant-derived extracts and compounds on the activity of mushroom and mammalian tyrosinases may vary significantly [49,50].

**Figure 5:**
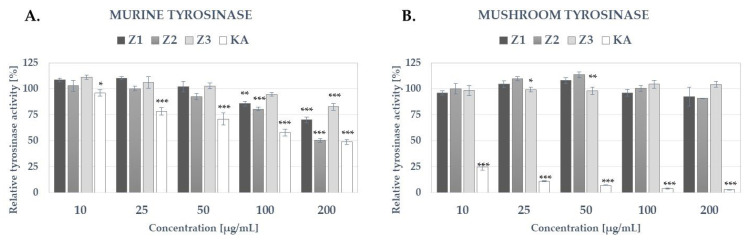
The influence of Z1, Z2 and Z3 extracts from *Z. bugeana* on the activity of murine (**A**) and mushroom (**B**) tyrosinase; histograms show mean tyrosinase activity ±SD, * *p* < 0.05, ** *p* < 0.01, *** *p* < 0.001; KA—kojic acid.

As shown in Figure 5, none of the analyzed *Ziziphora* extracts significantly inhibited mushroom tyrosinase up to the concentration of 200 µg/mL (Figure 5B). Extract Z2 slightly increased the activity of this enzyme at 25 and 50 µg/mL. In respect of the murine tyrosinase all *Ziziphora* extracts showed significant, dose-dependent inhibitory potential (Figure 5A). The most potent inhibitor of murine tyrosinase was extracts Z2, decreasing the activity of tyrosinase by 50% at 200 µg/mL which was comparable with the inhibitory activity of kojic acid (KA), a tyrosinase inhibitor widely used in skin lightening cosmetics [49]. Extracts Z3 showed the lowest activity, significantly decreasing the activity of tyrosinase only at the highest analyzed concentration (200 µg/mL). To our knowledge, this is the first study investigating the effect of *Ziziphora* spp. extract on the activity of mammalian tyrosinase.

Several phytochemicals identified in *Ziziphora* extracts were described in scientific literature as effective mushroom tyrosinase inhibitors, including acetophenone [51] identified in *Z. tenuior* [52] and cuminyl aldehyde (syn. cumaldehyde) [53] from *Z. clinopodioides* subsp. *rigida* [54]. The last compound was also shown to suppress melanin synthesis in B16F10 murine melanoma cells [55].

*Z. clinopodioides* extracts were previously found to exhibit weak tyrosinase inhibitory potential. The extracts from the overground parts of the plant exhibited weak inhibitory potential against the enzyme at the concentration of 200 μg/mL with 8.60 ± 0.87% inhibition compared to kojic acid (KA) for whom the inhibition percentage was calculated as 95.26 ± 0.23% [3].

Mushroom tyrosinase activity was also analyzed by Tomczyk and co-workers in respect of *Z. taurica* extracts [45]. The IC_50_ values for aqueous, ethyl acetate and methanolic extracts were 2.29 ± 0.13, 1.37 ± 0.07 and 1.46 ± 0.06 mg/mL, respectively. These values suggest that *Z. bungeana* extracts Z1, Z2, and Z3 might be effective against mushroom tyrosinase, but at higher concentrations than tested in this study.

### 2.5. Cytotoxic Activity

*Ziziphora* spp. were shown to contain several compounds with broad range of anticancer activities, including pulegone, menthol, menthone, cineole, piperitone, isomenthol, and curcumin [10]. However, only a few recent studies described the anti-cancer potential of whole *Ziziphora* extracts and EOs [56,57,58].

**Figure 6:**
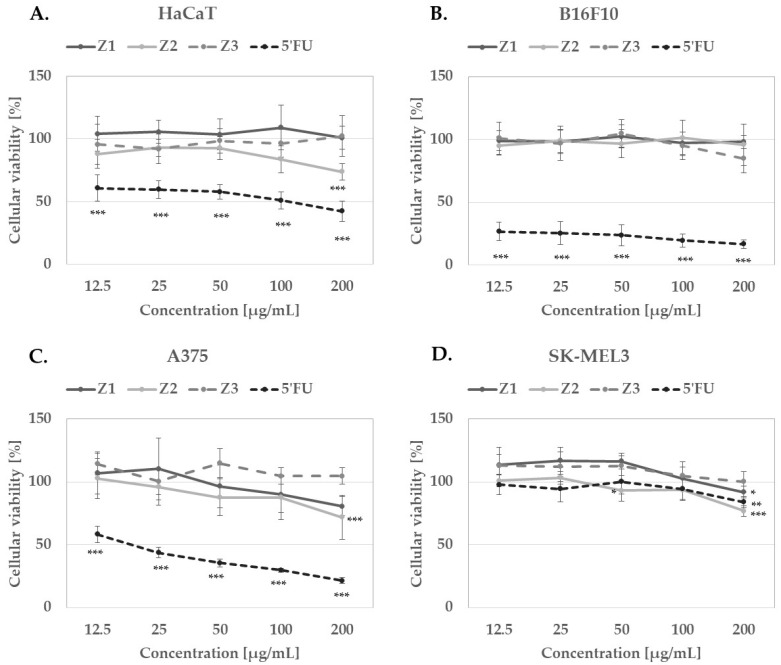
In vitro cytotoxicity of Z1, Z2 and Z3 *Z. bungeana* extracts on human keratinocytes HaCaT (**A**), murine melanoma B16F10 (**B**) and human melanoma cell lines A375 (**C**) and SK-MEL3 (**D**) following 48 h culture; graphs show mean viability of the cells ± SD in comparison with appropriate solvent controls; * *p* < 0.05, ** *p* < 0.01, *** *p* < 0.001.

In this study, the authors focused on the assessment of *Z. bungeana* cytotoxic effect on human and murine melanoma cells (Figure 6B–D) in comparison with a known chemotherapeutic agent, 5′fluorouracil (5′FU). Human keratinocytes HaCaT served as noncancerous control cells (Figure 5A).

Extract Z1 at 200 µg/mL was slightly cytotoxic for human melanoma A375 cell line, reducing the number of viable cells by ca. 20%. It was not cytotoxic for HaCaT keratinocytes, B16F10, and SKMEL-3 melanoma cells. Extract Z2 at 200 µg/mL significantly reduced the number of viable A375 and SK-MEL3 melanoma cells by ca 28% and 23%. However, it showed comparable cytotoxicity towards HaCaT keratinocytes. Extract Z3 was cytotoxic only for B16F10 murine melanoma cells, reducing their viability by 15% at 200 µg/mL.

The cytotoxicity of *Ziziphora* spp. Extracts towards murine and human melanoma cells as well as human noncancerous skin cells has not been described in the scientific literature to date. Several compounds found in *Ziziphora* preparations, such as extracts and EO, including linalool and α-terpineol (*Z. clinopodioides*), carvacrol (*Z. tenuior, Z. clinopodioides*), thymol (*Z. tenuior*), and terpinen-4-ol (*Z. clinopodioides*), are known to induce apoptosis in melanoma cell lines [11]. In the study of Yousefbeyk and co-investigators [59] *Z. clinopoides* n-hexane extract that was found rich in pulegone, menthone and menthol exhibited strong cytotoxic activity against K-562 and T-47D cell lines with EC_50_ values of 80 ± 2.56 μg/mL and 77.41 ± 12.89, respectively. Interestingly, more polar fractions did not show cytotoxic effects.

Available scientific data on the in vitro cytotoxic effect of *Ziziphora* spp. preparations were obtained using EOs. Azimi and co-workers showed that the EO from *Z. tenuior* induces apoptosis in human colorectal cancer cells HT-29 in a concentration range of 50–200 µg/mL. The apoptotic effect was mediated by increased caspase 3 and 9 expression at mRNA and protein levels and decreased levels of Bcl-2 [56]. Ghavan et al. showed that *Z. clinopodioides* subsp. *rigida* EO is cytotoxic for human ovarian cancer cells (OVCAR-3) [60].

### 2.6. The Hemolytic Activity Assay (Toxicity towards Erythrocytes)

**Figure 7:**
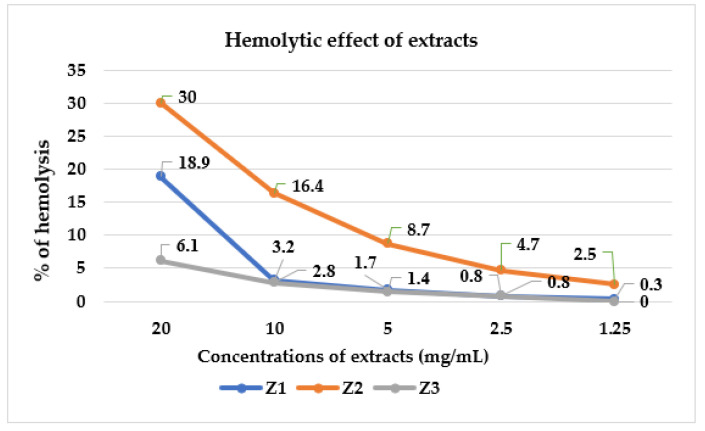
Hemolytic effect (% of hemolysis) of the studied extracts from *Z. bungeana*.

In the present studies, the toxicity of Z1–Z3 extracts from *Z. bungeana* towards red blood cells was calculated in vitro hemolytic assay. The erythrocyte model (erythrocyte lysis assay; ELA) was used to analyzed their effect on cell membrane [60]. The results revealed that studied extracts exhibit negligible toxicity as compared to the positive control Triton X-100 (100% erythrocyte lysis). As presented in Figure 7, hemolytic activity of each extract was related to their concentration. The highest concentrations of the studied extracts (20 mg/mL) showed some hemolytic activity in the range 6.1–30%. In turn, their concentrations that did not exert any hemolytic effect ranged from 1.25 to 2.5 mg/mL and the percentage of lysed red blood cells of 0–4.5 was within the permissible limit of 5% hemolysis [61]. The Z1 and Z3 extracts exhibited lower hemolytic activity (0–18.9%) than Z2 (2.5–30%) and did not affect the stability of the erythrocyte membrane. Data obtained using ELA confirm that antimicrobial effect, especially against Gram-positive bacteria (MIC = 1.25–5 mg/mL), was observed at non-cytotoxic concentrations of extracts from *Z. bungeana*.

The erythrocyte model presents general indication of membrane toxicity. The red blood cell membrane shows similarity to other cell membranes. Hemolysis is due to erythrocyte cells destruction resulting from lysis of the membrane lipid bilayer [60,61]. The obtained data using ELA confirm antibacterial activity of Z1–Z3 extracts at their non-cytotoxic concentrations (MIC = 1.25–5 mg/mL) against staphylococci, micrococci, and some bacilli. Therefore, it seems practical to use these extracts in the future in the prevention and treatment of infections caused by selected microorganisms, mainly Gram-positive bacteria.

### 2.7. Chemometric Assessment

**Figure 8:**
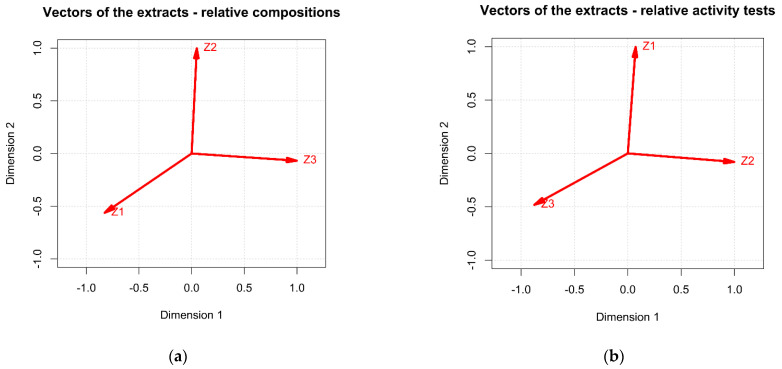
Relations of the **Z1**–**Z3** vectors in the space of the first two principal components (subjected to VARIMAX rotation), regarding their relative compositions (**a**) and relative biological activities (**b**).

Principal component analysis (PCA) was conducted separately for the Z1–Z3 relative compositions (C_r**el**_) and the Z1–Z3 relative activities (A**_rel_**) (See Tables S2 and S3 in the Supplementary File), whereas the extracts were treated as vectors defined on C**_rel_** or A**_rel_** values. The resulting spaces were two-dimensional, since in both cases the first two principal components extracted almost 100% of the information (expressed as variance) of studied ensembles. In the case of the C**_rel_** system (Figure 8A), the relative composition of the Z3 and Z1 extracts were reversely correlated, constituting the first dimension of the studied dataset, whereas the Z2 extract exhibited rather unique proportions of the selected eleven analyzed compounds (named in the Table 1 as **1**, **2**, **4**, **6**, **9**, **10**, **11**, **13**, **15**, **23**, **26**—see Table S2 in the Supplementary File), defining the second dimension of the vector space. The obtained conclusions are logical, as dichloromethane is characterized by a much lower polarity than ethanol and water, and that is why the extracts obtained using dichloromethane can show a different fingerprint from alcoholic or water ones. In the case of A**_rel_** dataset (Figure 8B), the relative biological activities of Z2 and Z3 extracts were reversely correlated (Dimension 1), while the Z1 extract exhibited different properties, possibly thanks to other metabolites, e.g., peptides, sugars, or proteins whose identity was not analyzed in this study, solely explaining the second dimension of the studied space. 

**Figure 9:**
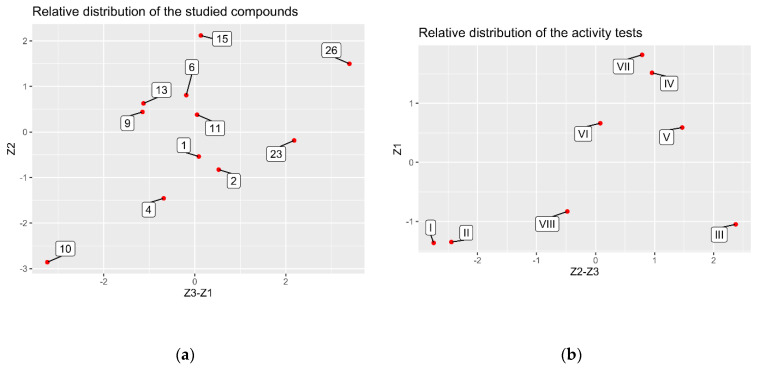
Linear maps of the selected compounds in the space of the first two principal components (subjected to VARIMAX rotation), regarding their relative compositions (**a**) and relative biological activities (**b**).

The linear maps of the studied compounds (Figure 9A) and the activity tests (Figure 9B) were presented in the same spaces as the respective linear maps of the Z1–Z3 vectors in Figure 8. In the case of C_rel_ ensemble, the relative amounts of the **1**, **2**, **6**, **9**, **11**, and **13** compounds were very similar for all three extracts. Z3 clearly excelled in the relative amounts of **23** and **26**, whereas it was very low on **10**′s concentration. On the contrary, Z1 contained impressive amounts of **10** while lacking **23** and **26**. Z2 was quite low in 4 and **10** yet exhibited higher-than-average amounts of **15** and **26**. While taking into account the relative values of activity tests, Z3 excelled with the B16F10 cell line (I and II), on the contrary to Z1. Moreover, Z2 was quite good at IV (A375 cell line) and V (SKMEL-3 cell line), yet toxic to HaCaT cells (III), whereas Z1 exhibited poor activity at VIII (SOD assay).

While comparing the above with the distribution of the relative compositions of the eleven analyzed compounds (named in Table 1 as **1**, **2**, **4**, **6**, **9**, **10**, **11**, **13**, **15**, **23**, **26**) within the Z1–Z3 extracts, one might conclude that eventual toxic effects towards HaCat cell line (III) at the highest dose, exhibited solely by Z2, could result from the presence of very high, relative amounts of the compounds **15** and **26**. These findings may be due to the fact, that the analyzed concentration used in the calculations was high and exceeded safe doses for both diosmin and ursolic acid, respectively. In the meantime, Z2 utterly failed at the tests I and II (B16F10 cell line), similarly to Z1. Since Z3 succeeded at I and II, while it was also rich with the compounds **23** and **26**, the good result at I and II could be directly associated with high relative amounts of **23** (acacetin). Finally, Z1 extract did relatively well at the tests IV (A375 cell line) and VII (mushroom tyrosinase assay), while it exhibited high amounts of a ziziphoroside isomer 2 (**10**). Possibly, its presence influences the total activity of the extract. Previously, other species of *Ziziphora* were proven to inhibit tyrosinase [10]. On the basis of the resulting images, no other compounds could be related to the biological activities of Z1–Z3 extracts in a straightforward manner.

## 3. Material and Methods

### 3.1. Materials

#### 3.1.1. Plant Material

The aerial parts of *Ziziphora bungeana* Lam. were collected in the summer of 2021 in the flowering stage in the Turkestan region of the Republic of Kazakhstan and identified by the Institute of Botany and Phytointroduction, Science Committee, Ministry of Education and Science of the Republic of Kazakhstan. A voucher sample (№01-05/337 from 5 October 2021) has been deposited in the herbarium of the Institute of Botany and Phytointroduction, Almaty, Republic of Kazakhstan.

#### 3.1.2. Microorganisms

The reference strains of microorganisms from American Type Culture Collection (ATCC) (Manassas, VA, USA) were used in the study. The representative Gram-positive bacteria were: *Staphylococcus aureus* ATCC 29,213 (Methicillin Susceptible *Staphylococcus aureus*—MSSA), *Staphylococcus aureus* ATCC 43,300 (Methicillin Resistant *Staphylococcus aureus*—MRSA), *Staphylococcus epidermidis* ATCC 12228, *Enterococcus faecalis* ATCC 29212, *Micrococcus luteus* ATCC 10240, *Bacillus subtilis* ATCC 6633 and *Bacillus cereus* ATCC 10876), while those of Gram-negative bacteria: *Escherichia coli* ATCC 25922, *Klebsiella pneumoniae* ATCC 13883, *Salmonella typhimurium* ATCC 14028, *Pseudomonas aeruginosa* ATCC 9027 and *Bordetella bronchiseptica* ATCC 4617. Moreover, the fungi belonging to yeasts: *Candida albicans* ATCC 10231, *Candida albicans* ATCC 2091, *Candida parapsilosis* ATCC 22019, *Candida glabrata* ATCC 90030, and *Candida krusei* ATCC 14,243 were used.

### 3.2. Methods

#### 3.2.1. Extraction Procedure

First, the aerial parts of the plant were powdered using an electric mill (type WZ-1, ZBPP, Poland). Next, 10 g portions of the aerial parts of the plant were divided into three parts to provide three extracts using the following extracting solvents—water (Z1), dichloromethane (Z2) and 96% ethanol (Z3). After adding 50 mL of solvents, the extraction was performed three times, 30 min each, at room temperature using an ultrasonic bath with no heating. Then, the extracts were centrifuged at 3500 rpm for 10 min, filtered through a nylon syringe filter (pore diameter 0.22 µm), and evaporated in the weighted vials using the Eppendorf Concentrator Plus (Hamburg, Germany) at the temperature of 45 °C. Weighted samples were kept in the freezer before chromatographic studies and bioactivity evaluations.

#### 3.2.2. The HPLC-ESI-QTOF-MS/MS Analysis

Compositional studies of *Z. bungeana* extracts were performed using an HPLC-MS platform produced by Agilent Technologies (Santa Clara, CA, USA) which was composed of an HPLC chromatograph equipped in a binary pump (G1312C), a degasser (G1322A), an autosampler (G1329B), a photodiode array detector (DAD) (G1315D), and a QTOF-MS/MS mass spectrometer (G6530B).

The extracts’ constituents were separated in gradient method composed of 0.1% formic acid (solvent A) and acetonitrile with the addition of 0.1% formic acid (solvent B) in the following program: 0 min: 10% B, 10 min: 20% B, 15 min: 40% B, 17–22 min: 95% B, 22.10 min: 10% B. The run lasted 30 min, the flow rate was set at 0.200 mL/min and the injection volume was set at 5 µL, and the concentration of the extracts was 10 mg/mL. Chromatographic separation was performed on the RP-18 chromatographic column (dimensions: 150 mm × 2.1 mm; dp = 3.5 µm) (Zorbax Eclipse Plus by Agilent Technologies, Santa Clara, CA, USA).

The detection on the mass spectrometer was achieved in the following settings, using both negative and positive ionization mode: *m*/*z* range of 100–1700 Da, capillary voltage of 3000 V, gas and sheath gas temperatures of 275 and 325 °C, gas flows of 12 L/min, respectively, fragmentation voltage of 110 V, skimmer voltage of 65 V, and collision energies of 10 and 20 V. In the used method, the MS/MS spectra were recorded for the two most intense peaks per scan The structure determination was based on the fragmentation spectra, literature data, retention times, and open databases (Metlin).

#### 3.2.3. In Vitro Antimicrobial Activity Assay

The three extracts Z1–Z3 from *Ziziphora bungeana* were investigated in vitro for antibacterial and antifungal activities. In these studies, the broth microdilution was used. The tests were performed in accordance with the guidelines of the European Committee on Antimicrobial Susceptibility Testing (EUCAST) [14,62,63] and Clinical and Laboratory Standards [64,65]. The used microbial cultures were first subcultured and on nutrient agar (for bacteria) or Sabouraud agar (for fungi) (BioMaxima S.A., Lublin, Poland) and incubated at 35 °C for 18–24 h. Microbial suspensions were prepared in sterile saline (0.85% NaCl) with an optical density of 0.5 McFarland standard scale (1.5 × 10^8^ CFU/mL (CFU—Colony Forming Units/mL) for bacteria and 5 × 10^6^ CFU/mL for yeasts). Samples containing examined extracts were first dissolved in dimethyl sulfoxide (DMSO) to the concentration of 200 mg/mL. The minimal inhibitory concentration (MIC) of these extracts was evaluated by the microdilution broth method in 96-well polystyrene plates. In this study, two-fold dilutions of the extracts in selective broth, Mueller-Hinton (MH) (BioMaxima S.A., Lublin, Poland) for bacteria and RPMI (Roswell Park Memorial Institute) 1640 with MOPS (3-(*N*-Morpholino)propanesulfonic acid) (Sigma-Aldrich Chemicals, St. Louis, MO, USA), were performed. The final concentrations of extracts (diluted in broth) ranged from 20 to 0.156 mg/mL.

Next, the bacterial or fungal suspensions were introduced into each well of the microplate to obtain final density of 1.5 × 10^6^ CFU/mL for bacteria and 5 × 10^4^ CFU/mL for yeasts. After 18–24 h incubation at 35 °C, the MIC value was assessed in the BioTek spectrophotometer (Biokom, Janki, Poland) as the minimal concentration of the samples that showed complete microbial growth inhibition. The inhibition of bacterial and fungal growth was assessed by comparison with control cultures in media without any sample tested. Standard drugs: ciprofloxacin (antibacterial chemotherapeutic) and nystatin (antifungal antibiotic) (Sigma-Aldrich Chemicals, St. Louis, MO, USA) were used as reference substances. Appropriate DMSO, sterile, and growth controls were prepared. The media with and without tested extracts/DMSO were used as controls [13,62,63,64,65,66,67].

Subsequently, minimal bactericidal concentration (MBC) or minimal fungicidal concentration (MFC) values of extracts were determined by transferring the cultures from each MIC determination well to the appropriate solid medium. After incubation, the lowest concentrations of extracts with no visible bacterial or fungal growth were evaluated as MBC or MFC. All the experiments were repeated three times as independent assays, and representative data are presented [13,62,63,64,65,66]. The MBC/MIC or MFC/MIC ratios were calculated in order to determine bactericidal/fungicidal (MBC/MIC ≤ 4, MFC/MIC ≤ 4) or bacteriostatic/fungistatic (MBC/MIC > 4, MFC/MIC > 4) effect of the tested extracts [65].

#### 3.2.4. Total Antimicrobial Activity (TAA) Assay

The total antibacterial activity or total antifungal activity (TAA) of each of Z1, Z2, and Z3 extracts from *Z. bungeana* was obtained by dividing the quantity extracted from one gram of each plant extract by the MIC value. TAA was calculated following a standard formula:

(1) $$TAA\;\left(mL/g\right)=\frac{Mass\;of\;extract\;from\;1\;gram\;of\;powder\;\left(mg\;per\;gram\right)\;\;\;\;\;}{MIC\;\;\left(mg\;per\;mL\right)}$$

The total antibacterial activity (*TAA*) is a function of the extraction yield in milligram per 1 g of plant material and the minimal inhibitory concentration (*MIC*), expressed in milliliter per gram (mL/g). *TAA* indicates the volume of water or solvent, when added to 1 g of the extract, that will still inhibit the growth of the pathogen [68,69,70].

#### 3.2.5. Antioxidant Activity

##### DPPH Scavenging Assay

The DPPH radical scavenging assay was performed as described by Matejic et al. [71]. Briefly, 100 µL of Z1, Z2 or Z3 diluted extracts (0.48–1000 µg/mL) was mixed with equal volume DPPH working solution (25 mM DPPH in 99.9% methanol; A540 ≈ 1). 100 µL of the solvent mixed with 100 µL DPPH served as a control sample. After 20 min incubation at RT in darkness, the absorbance of the samples was measured at λ = 540 nm using a FilterMax F5 microplate reader (Molecular Devices, San Jose, CA, USA). Obtained values of measurements were corrected by the absorbance values of the samples without DPPH. The percentage of DPPH radical scavenging was calculated based on the equation:

% of DPPH˙ scavenging = [1 − (Abs(S)/Abs(C))] × 100%(2)

where Abs(S) is the absorbance of the sample and Abs(C) is the absorbance of the control sample (DPPH + solvent).

Obtained results were used to calculated EC_50_ values defined as the concentration of dried extract/fraction that is required to scavenge 50% of the DPPH radical activity.

##### ABTS Scavenging Assay

ABTS radical scavenging assay was performed according to Re and co-workers [72] with some modifications. Briefly, 135 µL of ABTS working solution (7 mM ABTS in 2.45 mM K_2_S_2_O_8_ diluted in distilled H_2_O up to A405 ≈ 1) was mixed with 15 µL of Z1, Z2 or Z3 diluted extract (0.48–1000 µg/mL) or solvent control. Following 15 min incubation at RT in darkness, the absorbance of the samples was measured at λ = 405 nm using a microplate reader (FilterMax F5 Molecular Devices, USA). The obtained values were corrected by the absorbance value of the sample without ABTS. The percentage of ABTS radical scavenging was calculated based on the equation:

% of ABTS scavenging = [1 − (Abs(S)/Abs(C))] × 100%(3)

where Abs(S) is the absorbance of the extract and Abs(C) is the absorbance of the control sample (ABTS + solvent).

Obtained results were used to calculated EC_50_ values defined as the concentration of dried extract/fraction that is required to scavenge 50% of the ABTS radical activity.

##### SOD Inhibitory Assay

The influence of Z1, Z2, and Z3 extracts on the activity of superoxide dismutase (SOD) was measured using SOD Determination Kit (cat. No. 19160, Sigma Aldrich, Merck, Darmstadt, Germany), according to manufacturer’s instructions.

#### 3.2.6. Tyrosinase Inhibitory Activity

The tyrosinase inhibitory activity of Z1, Z2, Z3 extracts was compared using commercially available mushroom tyrosinase (Sigma Aldrich) and murine tyrosinase contained in the lysate of B16F10 murine melanoma cells (ATCC CRL-6475; LGC Standards, Łomianki, Poland), prepared as previously described [73]. 

Mushroom tyrosinase activity assay was performed according to the protocol described by Uchida and co-workers [74] For this analysis, 120 μL phosphate buffer (100 mM, pH = 6.8) was mixed with 20 μL of diluted extracts (final concentrations 10–200 µg/mL) and 20 μL of mushroom tyrosinase (500 U/mL) and pre-incubated at room temperature for 10 min. Following the addition of 40 μL 4 mM L-DOPA, the samples were incubated for another 20 min at RT. 

The activity of murine tyrosinase was assessed by Incubating the volume of B16F10 cell lysate containing 20 µg protein with 20 µL of diluted extracts (final concentrations 10–200 µg/mL), 40 µL 4 mM l-DOPA and 100 mM phosphate buffer pH 6.8 (up to 200 µL). The reaction was carried out for 4 h at 37 °C. Control samples (100% tyrosinase activity) for both assays contained an appropriate volume of the solvent instead of the extract. In both assays, the dopachrome formation was measured spectrophotometrically at λ = 450 nm using FilterMax F5 microplate reader (Molecular Devices, USA). The obtained values were corrected by the absorbance value of the extracts without mushroom or murine tyrosinase and l-DOPA. Each sample was analyzed in 3 independent repetitions. Kojic acid was used as a known tyrosinase inhibitor control.

#### 3.2.7. In Vitro Cytotoxicity Assay

The cytotoxicity of Z1, Z2 and Z3 extracts was established by Neutral Red Uptake Test, as described by Repetto et al. [75] using human immortalized keratinocytes HaCaT (CLS Cell Lines Service GmbH, Eppelheim, Germany) [76], murine melanoma B16F10 (ATCC CRL-6475) and human melanoma A375 (ATCC CRL-1619) and SK-MEL3 (ATCC HTB-69) (LGC Standards, Łomianki, Poland). All cell lines were maintained in Dulbecco’s modified Eagle’s medium (DMEM)/high glucose supplemented with 10% fetal bovine serum (FBS, Pan Biotech, Aidenbach, Germany) at 37 °C in a humidified atmosphere with 5% CO_2_. For the experiments 3000 cells were plated per well onto a 96-well plate and grown overnight. Then, the cells were treated with various concentrations of Z1, Z2, or Z3 extracts (12.5–200 µg/mL) or an equal volume of the solvent control. Following 48 h of culture, the cells were incubated for 3 h in DMEM containing 1% FBS and 33 µg/mL neutral red, following by washing in PBS and lysis using acidified ethanol solution (50% *v/v* ethanol, 1% *v/v* acetic acid). The absorbance of the released neutral red was measured using FilterMax F5 microplate reader (Molecular Devices, San Jose, CA, USA) at λ= 540 nm. The mean measurement value for the lysate from control cells was set as 100% cellular viability and used to calculate the percentage of viable cells following extracts treatment. 

#### 3.2.8. Toxicity to Erythrocyte Assay

The erythrocyte lysis assay (ELA) was performed to study the toxicity of the extracts Z1, Z2 and Z3 from *Ziziphora bungeana* on red blood cells. In the first, erythrocytes were harvested from 5.0 mL fresh sheep blood (BioMaxima S.A., Poland) by centrifugation for 10 min at 1000× *g* and washed with 0.85% NaCl. Subsequently, 2% erythrocyte suspension was prepared in sterile phosphate buffer saline and in a volume of 100 μL was added to each well of a 96-well microtiter plate. The serial dilutions of these extracts ranging from 0.01 to 20 mg/mL were performed. To estimate the relative hemolytic potential of Z1, Z2, and Z3, the appropriate controls, i.e., 100% erythrocyte lysis using 4% Triton X-100 (Pol-Aura, Różnowo, Poland) and 0% lysis in saline solution, were used. Plates with samples were incubated for 1 h at 37 °C, then centrifuged for 10 min at 1000× *g* to separate the unlysed erythrocytes, and subsequently, the supernatant was transferred to a new plate. The absorbance was measured spectrophotometrically at 450 nm. The ELA represents an advantageous bioassay, because the lytic response can be measured photometrically by the amount of released hemoglobin. The hemolysis percentage was calculated according to the equation: % hemolysis = [(A450 of tested extract treated sample-A450 of buffer treated sample)/(A450 of 4% Triton X-100 treated samples-A450 of buffer treated sample)] × 100 [60,77,78,79].

### 3.3. Chemometric Analysis

All the chemometric analyses and visualizations were performed using R v4.2.0 [80] programming language in RStudio [81] software with pracma [82], factoextra [83], matlib [84], and corrplot [85] packages installed. After the standard, formal decomposition of the covariance matrices was calculated for the C**_rel_** (relative compositions) and A**_rel_** (relative activities) autoscaled datasets, and two principal components (PCs) were considered relevant in both cases. After the selection of the relevant PCs, their vectors were rotated in space in order to maximize the values of correlation coefficients between the original variables and the two orthogonal factors using the VARIMAX algorithm. In every case, compound/activity test scores in the space of the resulting varivectors (dimensions) were calculated by multiplying the matrix of the autoscaled C**_rel_**/A**_rel_** dataset by the matrix of the original variables’ loadings in the space of the resulting varivectors.

## 4. Conclusions

The presented results show the significance of *Ziziphora bungeana* extracts in terms of their composition and bioactivity. Twenty-six secondary metabolites were identified in the prepared extracts from *Z. bungeana* in the HPLC-ESI-QTOF-MS/MS analysis, that belonged to flavonoids, phenolic acids, terpenes, and organic acids. The results of antimicrobial studies indicated that extracts Z1, Z2, and Z3 showed potential activity with bactericidal or fungicidal effects. Among reference microorganisms, Gram-positive bacteria strains *Staphylococcus* spp., *Micrococcus luteus*, followed by *Bacillus* spp. were the most susceptible to the tested extracts (3.347–20.202 mL/g) in comparison with Gram-negative bacteria and fungi. Spectrophotometric assays proved the strongest antiradical properties of Z3 (EC_50_ values of 15.00 ± 1.06 µg/mL and 13.21 ± 3.24 µg/mL for ABTS and DPPH assays, respectively) and a marked SOD stimulatory action (>90% SOD activity) for Z1. In the murine tyrosinase assay all *Ziziphora* extracts showed significant, dose-dependent whitening properties. The most potent inhibitor of murine tyrosinase was extract Z2, decreasing the activity of tyrosinase by 50% at 200 µg/mL which was comparable with the inhibitory activity of kojic acid. All extracts were slightly cytotoxic for melanoma cells. However, Z2 at the concentration of 200 µg/mL showed a comparable cytotoxicity towards HaCaT keratinocytes. Moreover, our data suggest that the extracts Z1 and Z3 are not toxic for HaCaT cell lines or for erythrocyte membranes at the tested concentrations, which gives hope for its potential internal and external administration. The chemometric analysis performed to deliver the connections between the composition and biological properties of the extracts confirmed a different identity of all three extracts. According to the obtained results, the presence of the ziziphoroside isomer could induce anti-tyrosinase properties to the highest extent, whereas the presence of a higher quantity of acacetin could increase the anticancer potential of an extract.

## Figures and Tables

**Table 1:** The list of tentatively identified compounds present in the analyzed samples that were obtained from the HPLC-ESI-QTOF-MS/MS analysis in positive and negative ionization modes (DBE—double bond equivalent, error—error of measurement in ppm, Ion.—ionisation type, Rt—retention time).

No	Ion.+/−	Rt [min]	Molecular Formula	*m*/*z* Theoretical	*m*/*z* Experimental	Error	DBE	MS/MS Spectrum	Proposed Compound	Distribution	References
1	−	3.03	C_16_H_18_O_9_	353.0878	353.0884	−1.68	8	191, 179, 173, 154	Chlorogenic acid	Z1, Z2, Z3	[13]
2	−	3.9	C_16_H_18_O_9_	353.0878	353.0880	−0.55	8	179, 191	Neochlorogenic acid	Z1, Z2, Z3	[13]
3	+	3.9	C_16_H_28_O_8_	349.1857	349.1851	1.71	3	281, 163	Schizonepetaside E	Z1, Z2, Z3	
4	−	4.4	C_16_H_18_O_9_	353.0878	353.0884	−1.68	8	191, 179, 173, 135, 155	(*Z*)-Chlorogenic acid	Z1, Z2, Z3	[13]
5	−	5.2	C_14_H_18_O_7_	297.098	297.1011	−10.48	6	ND	Picein	Traces Z3	[14]
6	−	6.5	C_9_H_8_O_4_	179.0350	179.0357	−3.99	6	135, 117, 107	Caffeic acid	Z1, Z2, Z3	[15]
7	−	7.7	C_11_H_12_O_4_	207.0663	207.0664	−0.57	6	192, 179, 174, 163, 135	Ethyl ester of caffeic acid	Z1, Z3	[16]
8	−	9.5	C_9_H_10_O_3_	165.0557	165.0540	10.34	5	ND	Apocynin	Traces: Z1, Z2, Z3	[17]
9	+	10.9	C_16_H_26_O_7_	331.1751 353.1571 (+Na)	331.1775 353.1607 (+Na)	−7.18 −10.3	4	201	Ziziphoroside isomer 1	Z1, Z2, Z3	[14]
10	+	12.9	C_16_H_26_O_7_	331.1751 353.1571 (+Na)	331.1761 353.1607 (+Na)	−2.94 −10.3	4	201, 147, 119	Ziziphoroside isomer 2	Z1, Z2, Z3	[14]
11	+	14.3	C_16_H_26_O_7_	331.1751 353.1571 (+Na)	331.1757 353.1609 (+Na)	−1.73 −10.86	4	201, 165, 147	Ziziphoroside isomer 3	Z1, Z2, Z3	[14]
12	−	18.5	C_21_H_18_O_11_	445.0776	445.0767	2.1	13	269, 175, 113	5,7,21-trihydroxyflavone-21- *O*-glucopyranoside	Z1, Z3	[18]
13	−	19.1	C_18_H_16_O_8_	359.0772	359.0780	−2.11	11	197, 179, 161, 135	Rosmarinic acid	Z1, Z2, Z3	[19]
14	+	19.8	C_10_H_14_O	151.1117	151.1129	−7.72	4	133, 123, 109, 105	Thymol	Z1, Z2, Z3	[20]
15	−	20.1	C_28_H_32_O_15_	607.1668	607.1671	−0.42	13	561, 253	Diosmin	Z1, Z2, Z3	[21]
16	−	20.4	C_28_H_32_O_14_	591.1719	591.1727	−1.3	13	ND	Linarin	Traces: Z1, Z2, Z3	[22]
17	−	20.9	C_15_H_10_O_7_	301.0354	301.0363	−3.06	11	ND	Quercetin	Z1, Z2, Z3 (traces)	[23]
18	−	21.1	C_15_H_10_O_6_	285.0405	285.0396	3.01	11	241, 151, 133	Luteolin	Z1, Z2, Z3	[24]
19	−	21.6	C_15_H_10_O_5_	269.0455	269.0462	2.03	11	225, 151	Apigenin	Z1, Z2, Z3	[25]
20		21.8	C_18_H_16_O_8_	359.0772	359.0772	0.11	11	344, 329	Thymonin	Z1, Z2, Z3	[26]
21	−	22.3	C_16_H_12_O_6_	299.0561	299.0555	2.04	11	284, 256, 165, 135	Diosmetin	Z1, Z2, Z3	[27]
22	+	22.6	C_10_H_14_O	151.1117	151.1135	−11.71	4	136, 123, 117, 105	Carvacrol	Z1, Z2, Z3	[28]
23	−	22.7	C_16_H_12_O_5_	283.0612	283.0620	−2.83	11	268, 240	Acacetin	Z1, Z2, Z3	[22]
24	−	23.0	C_30_H_48_O_3_	455.3531	455.3538	−1.6	7	455	Oleanolic acid	Z1, Z3	[22,29]
25	−	23.3	C_30_H_48_O_4_	471.3480	471.3479	0.18	7	337	Maslinic acid	Z1, Z2, Z3	[30]
26	−	24.0	C_30_H_48_O_3_	455.3531	455.3528	0.59	7	455	Ursolic acid	Z1, Z3	[31]

**Table 2:** The activity data of *Z. bungeana* extracts expressed as MIC (Minimum Inhibitory Concentration), MBC (Minimum Bactericidal Concentration) [mg/mL] and MBC/MIC value against the reference strains of microogranisms. (CIP—ciprofloxacin (MIC and MBC) [µg/mL]).

Species of Microorganism		Z1			Z2			Z3			CIP		
		MIC	MBC	MBC /MIC	MIC	MBC	MBC /MIC	MIC	MBC	MBC /MIC	MIC	MBC	MBC /MIC
**Gram-positive**	*Staphylococcus aureus* ATCC 29213	2.5	2.5	1	5	5	1	2.5	2.5	1	0.24	0.24	1
	*Staphylococcus aureus* ATCC 43300	2.5	2.5	1	2.5	5	2	2.5	5	2	0.24	0.24	1
	*Staphylococcus epidermidis* ATCC 12228	2.5	2.5	1	2.5	5	2	2.5	5	2	0.12	0.12	1
	*Micrococcus luteus* ATCC 10240	2.5	5	2	1.25	5	4	5	10	2	0.98	1.96	2
	*Bacillus subtilis* ATCC 6633	5	10	2	5	10	2	2.5	10	4	0.03	0.03	1
	*Bacillus cereus* ATCC 10876	10	10	1	5	10	2	5	10	2	0.06	0.12	2
**Gram-negative**	*Bordetella bronchiseptica* ATCC 4617	10	10	1	5	20	4	20	20	1	0.98	0.98	1
	*Klebsiella pneumoniae* ATCC 13883	20	20	1	10	20	2	20	20	1	0.12	0.12	1
	*Salmonella typhimurium* ATCC 14028	20	20	1	20	20	1	20	20	1	0.06	0.06	1
	*Escherichia coli* ATCC 25922	20	20	1	20	20	1	20	20	1	0.004	0.004	1
	*Pseudomonas aeruginosa* ATCC 9027	10	20	2	20	20	1	20	20	1	0.48	0.98	2

The representative (modal) data are presented. The sensitivity of the fungi belonging to *Candida* spp. to the tested extracts Z1–Z3 was similar to that of Gram-negative bacteria (MIC = 5–20 mg/mL and MFC = 20 mg/mL). *Candida parapsilosis* ATCC 22,019 was the most susceptible to Z2 and Z1 at MIC = 5 mg/mL and 10 mg/mL, respectively. Z2 showed also activity towards other *Candida* spp. with MIC = 10 mg/mL, except *Candida glabrata* ATCC 90,030 (MIC = 20 mg/mL). Moreover, the minimal concentrations of these extracts, which inhibited growth or killed these microorganisms were 20 mg/mL (Table 3).

**Table 3:** The activity data of *Z. bungeana* extracts expressed as MIC (Minimum Inhibitory Concentration), MFC (Minimum Fungicidal Concentration) [mg/mL] and MFC/MIC value against the reference strains of fungi (NYS —nystatin (MIC and MFC) [µg/mL]).

Species of Microorganism	Z1			Z2			Z3			NYS		
	MIC	MFC	MFC /MIC	MIC	MFC	MFC /MIC	MIC	MFC	MFC /MIC	MIC	MFC	MFC /MIC
*Candida albicans* ATCC 10231	20	20	1	10	20	2	20	20	1	0.48	0.48	1
*Candida albicans* ATCC 2091	20	20	1	10	20	2	20	20	1	0.24	0.24	1
*Candida parapsilosis* ATCC 22019	10	20	1	5	20	4	20	20	1	0.24	0.48	2
*Candida glabrata* ATCC 90030	20	20	1	20	20	1	20	20	1	0.24	0.48	2
*Candida krusei* ATCC 14243	20	20	1	10	20	2	20	20	1	0.24	0.24	1

The representative (modal) data are presented. As shown our results (Table 2 and Table 3), the most common MIC value of 20 mg/mL was found for Z1 (7 (43.75%) strains) and Z3 extracts (10 (62.5%) strains). In the case of Z2 extract, the values of MIC = 20 mg/mL and MIC = 10 mg/mL, occurred with the same frequency (25% each) against reference strains of microorganisms. The same frequency of MIC = 10 mg/mL was shown for Z1. MIC values of 5 mg/mL were shown for 5 (31.25%), 2 (12.5%) and 1 (6.25%) strains in the case of Z2, Z3 and Z1 extracts, respectively. The Z1 and Z3 inhibited the growth of microorganisms at the minimum concentration of 2.5 mg/mL (4 strains (25%) each). Additionally, Z2 inhibited the growth of 1 (6.25%) and 2 (12.5%) strains with MIC = 1.25 mg/mL and 2.5 mg/mL, respectively.

**Table 4:** The activity data of *Z. bungeana* extracts expressed as TAA (Total Antibacterial Activity or Total Antifungal Activity) [mL/g] against the reference strains of bacteria and fungi.

Species of Microorganism		TAA (mL/g)		
		Z1	Z2	Z3
**Gram-positive bacteria**	*Staphylococcus aureus* ATCC 29213	13.39 ± 0.0	6.74 ± 2.92	8.75 ± 0.0
	*Staphylococcus aureus* ATCC 43300	11.16 ± 3.86	8.42 ± 2.92	7.30 ± 2.53
	*Staphylococcus epidermidis* ATCC 12228	13.39 ± 0.0	10.10 ± 0.0	8.75 ± 0.0
	*Micrococcus luteus* ATCC 10240	13.39 ± 0.0	20.20 ± 0.0	5.84 ± 2.53
	*Bacillus subtilis* ATCC 6633	5.58 ± 1.93	5.05 ± 0.0	7.30 ± 2.53
	*Bacillus cereus* ATCC 10876	3.35 ± 0.0	4.21 ± 1.46	4.38 ± 0.0
**Gram-negative bacteria**	*Bordetella bronchiseptica* ATCC 4617	2.79 ± 0.97	4.21 ± 1.46	1.46 ± 0.63
	*Klebsiella pneumoniae* ATCC 13883	1.67 ± 0.0	2.53 ± 0.0	1.09 ± 0.0
	*Salmonella typhimurium* ATCC 14028	1.67 ± 0.0	1.68 ± 0.73	1.09 ± 0.0
	*Escherichia coli* ATCC 25922	2.23 ± 0.97	1.68 ± 0.73	1.46 ± 0.63
	*Pseudomonas aeruginosa* ATCC 9027	2.79 ± 0.97	1.26 ± 0.0	1.09 ± 0.0
**Fungi**	*Candida albicans* ATCC 10231	2.23 ± 0.97	2.53 ± 0.0	1.46 ± 0.63
	*Candida albicans* ATCC 2091	1.67 ± 0.0	2.53 ± 0.0	1.09 ± 0.0
	*Candida parapsilosis* ATCC 22019	3.35 ± 0.0	4.21 ± 1.46	1.46 ± 0.63
	*Candida glabrata* ATCC 90030	2.23 ± 0.97	1.68 ± 0.73	1.09 ± 0.0
	*Candida krusei* ATCC 14243	2.23 ± 0.97	2.10 ± 0.89	1.09 ± 0.0

## Data Availability

The obtained data are presented in the manuscript and supplementary File.

## Supplementary Materials

The following supporting information can be downloaded at: https://www.mdpi.com/article/10.3390/molecules27248994/s1. Figure S1: Fingerprints of the analyzed extracts in the negative ionization mode; Figure S2. Fingerprints of the analyzed extracts in the positive ionization modes; Table S1. MS/MS spectra of the identified compounds. Table S2: The relative compositions of the Z1–Z3 extracts. For the each studied compound (**1**–**26**), the HPLC peak areas representing the absolute amounts of a given compound within Z1, Z2 and Z3 extracts were rescaled, in order to sum to 1 in every row of the table below, expressing the relative compositions of the Z1–Z3 extracts; Table S3. The relative biological activities of the Z1–Z3 extracts. Biological activities represented by ‘survivability’ of cells/enzymes (S) were taken from Figure 6 for the Z1–Z3 extracts of concentrations equal to 200 ug/mL and used to calculate biological activity (A) using formula A = 100% − S. For each biological activity test (I–VIII), the values of A for the Z1, Z2 and Z3 extracts were rescaled in order to sum to 1 in every row of the table below.

## Sample Availability

Samples of the plant are available from the authors.
