# Supplementary material for: Multidirectional Characterization of Phytochemical Profile and Health-Promoting Effects of Ziziphora bungeana Juz. Extracts
Source: Molecules. 2022 Dec 16;27(24):8994. doi: 10.3390/molecules27248994 (PMC9788533; doi:10.3390/molecules27248994)
Supplement: Supplementary file 1 [file molecules-27-08994-s001.zip › molecules-2065328-supplementary.pdf]

SUPPLEMENTARY FILE

Figure S1. Fingerprints of the analyzed extracts in the negative ionization mode.

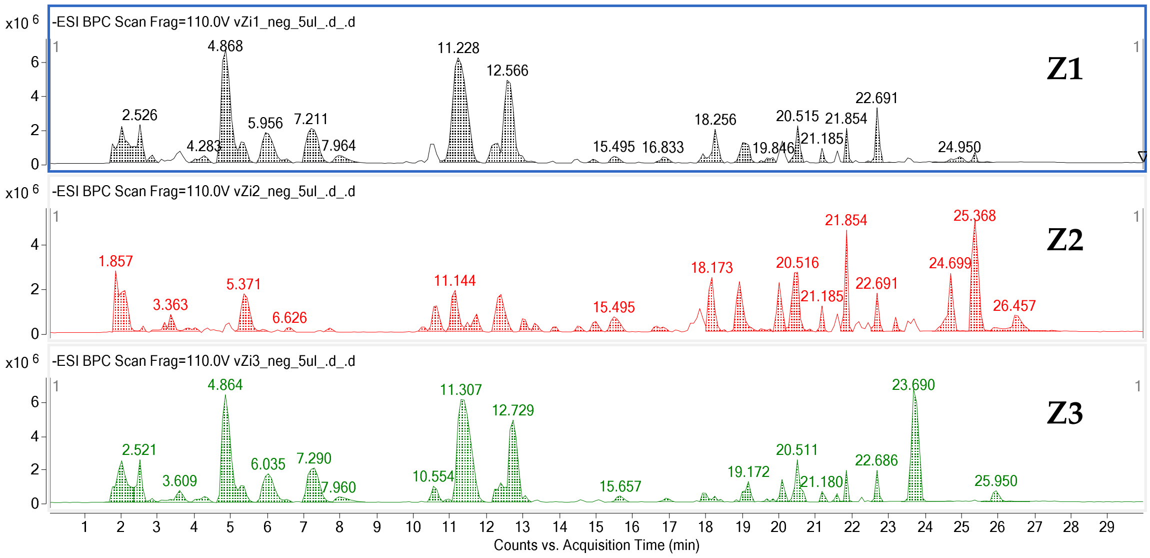

Figure S2. Fingerprints of the analyzed extracts in the positive ionization modes.

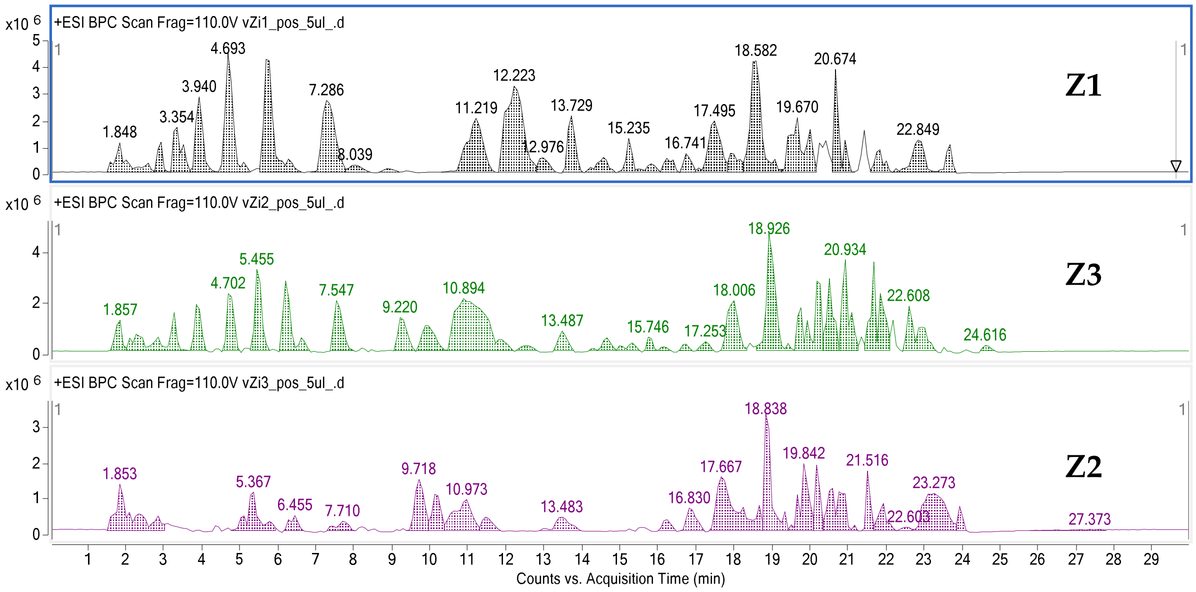

Table S1. MS/MS spectra of the identified compounds.

Compound	Spectrum
Apigenin	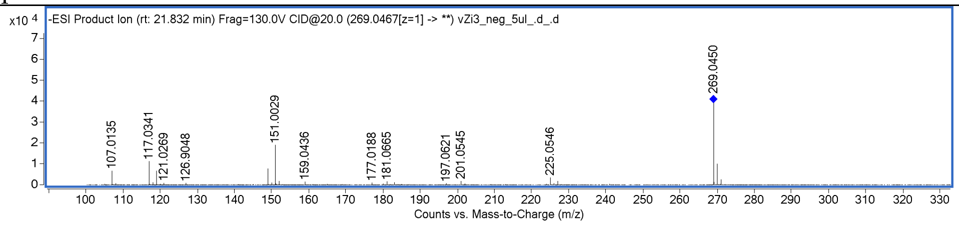
Chlorogenic acid	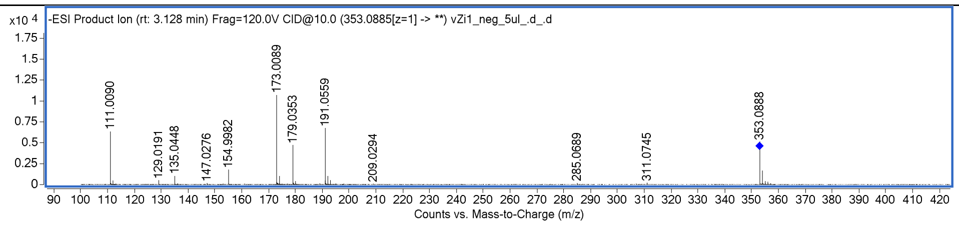
Neochlorogenic acid	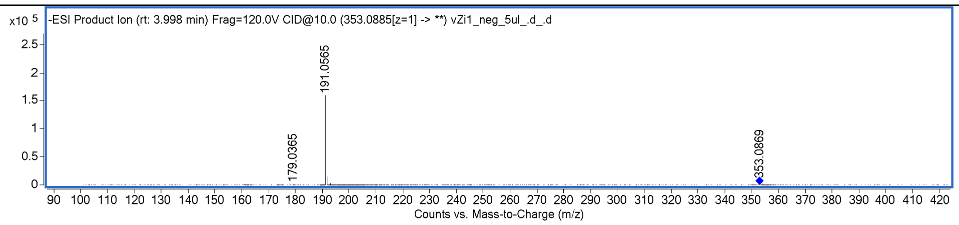
(*Z*)-Chlorogenic acid	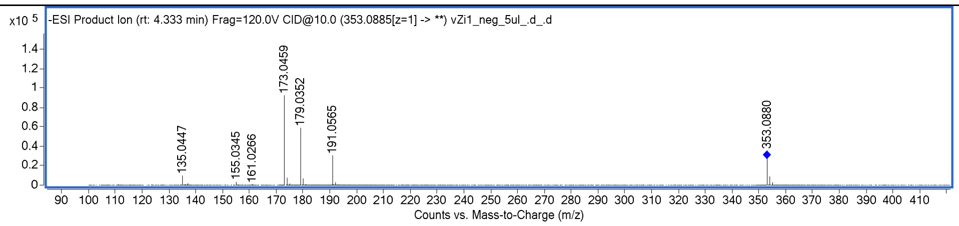
Luteolin	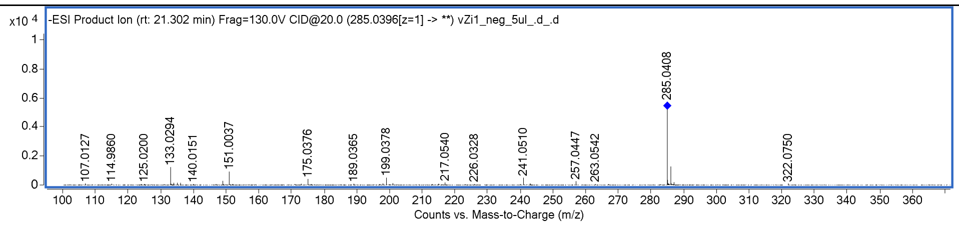
Thymonin and a dimer	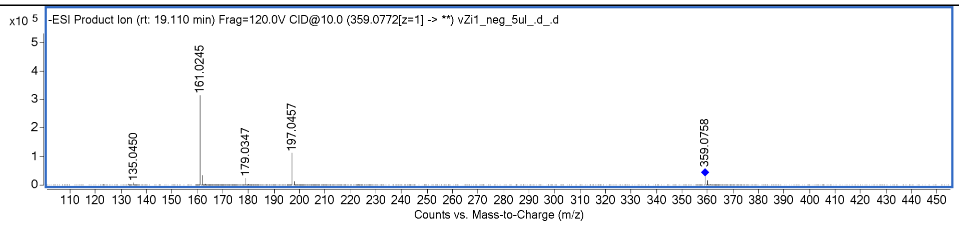

<table>
<tr><td></td><td>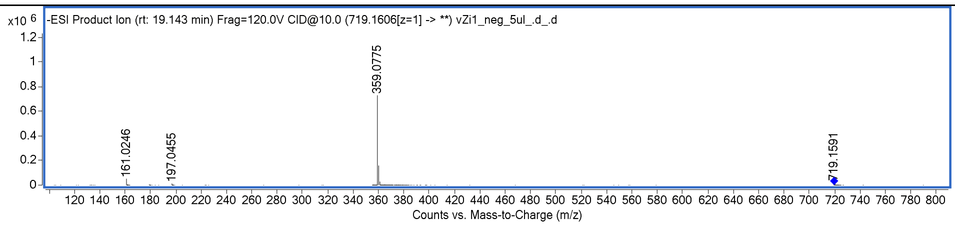
</td></tr>
<tr><td>Thymonin isomer</td><td>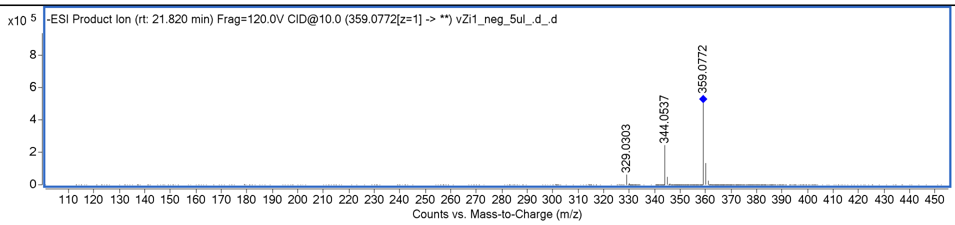
</td></tr>
<tr><td>Acacetin</td><td>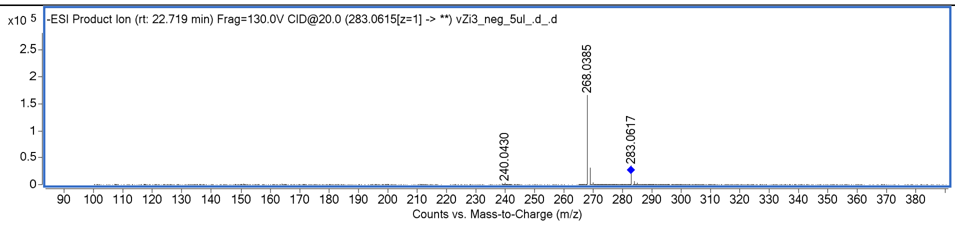
</td></tr>
<tr><td>Diosmetin</td><td>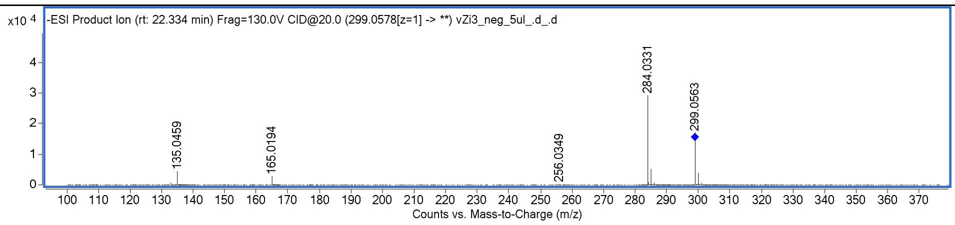
</td></tr>
<tr><td>Diosmetin</td><td>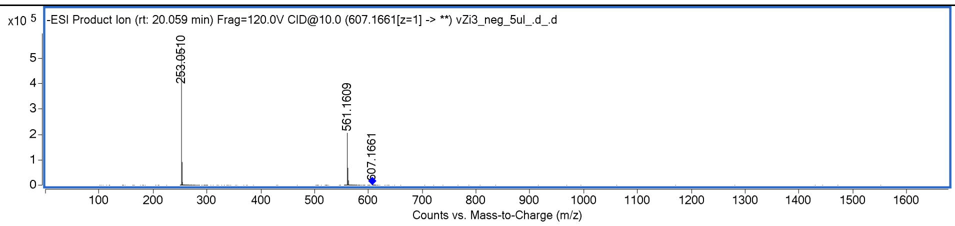
</td></tr>
<tr><td>5,7,21-trihydroxyflavone-21-O-glucopyranoside</td><td>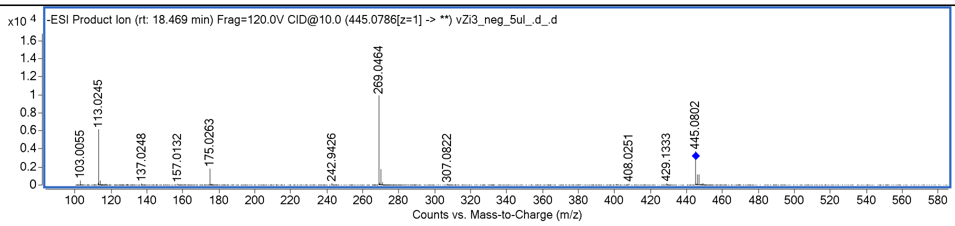
</td></tr>
</table>

Caffeic acid	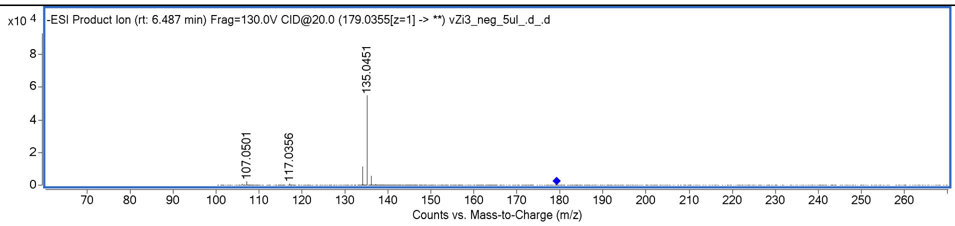
Ethyl ester of caffeic acid	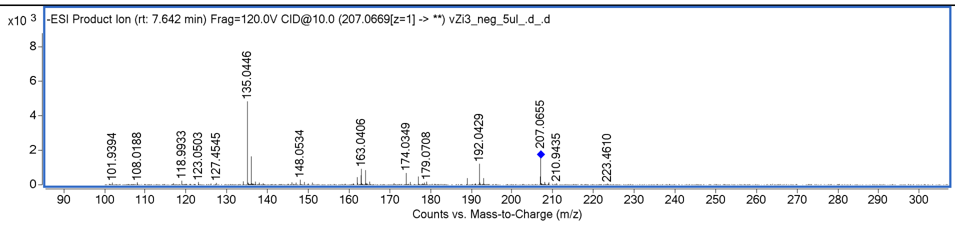
Oleanolic acid	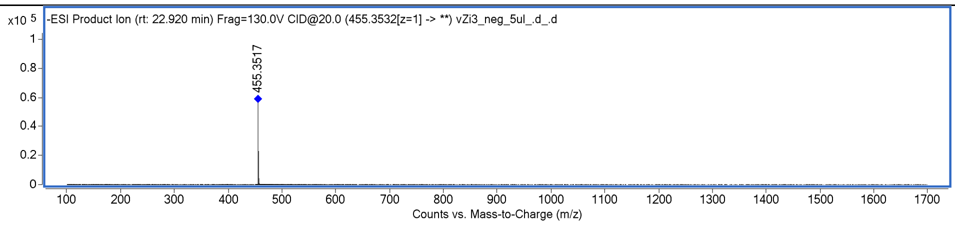
Ursolic acid	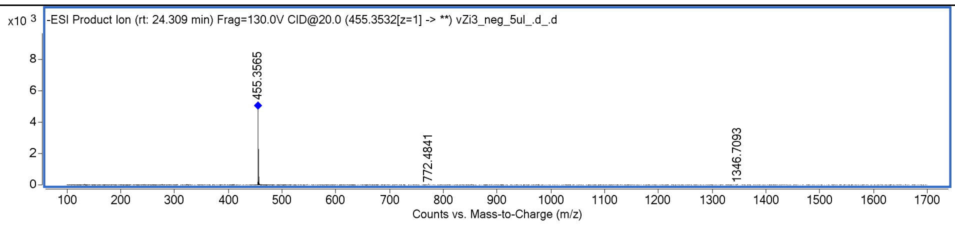
Maslinic acid	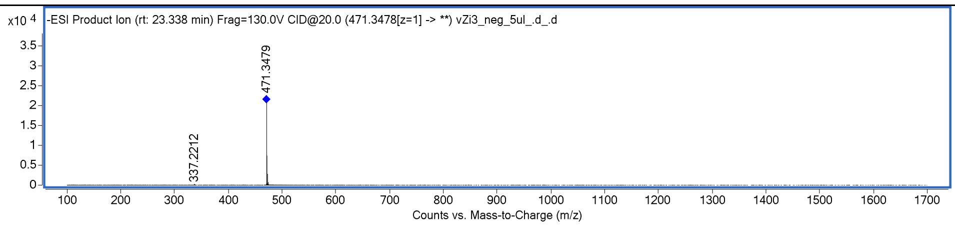
Thymol	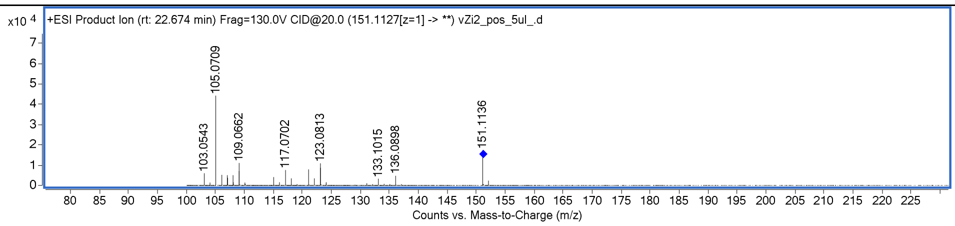

<table>
<tr><td>Carvacrol</td><td>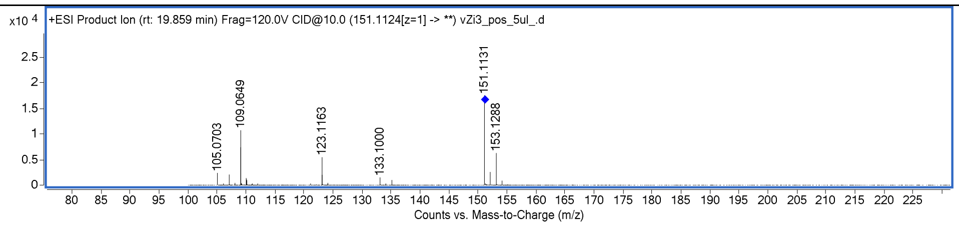
</td></tr>
<tr><td>Ziziphoroside A</td><td>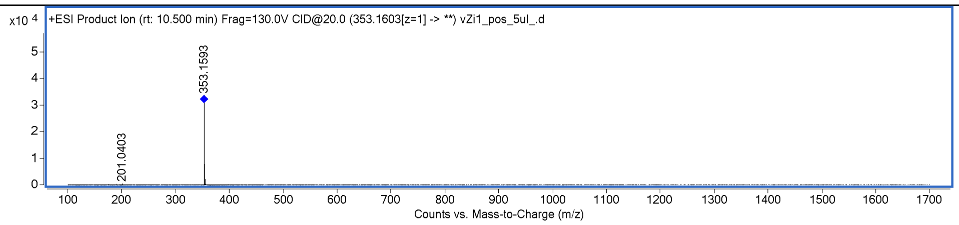
</td></tr>
<tr><td>Ziziphoroside B</td><td>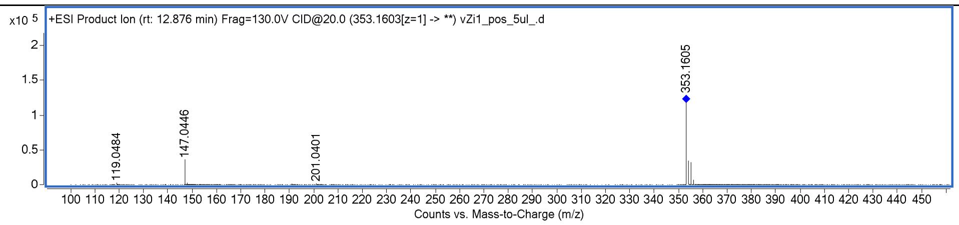
</td></tr>
<tr><td>Ziziphoroside C</td><td>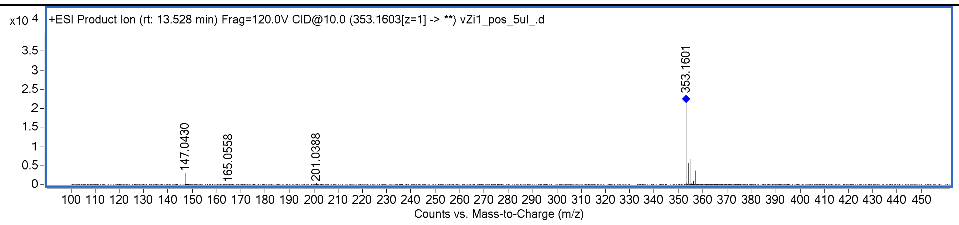
</td></tr>
<tr><td>Schizonepetaside E</td><td>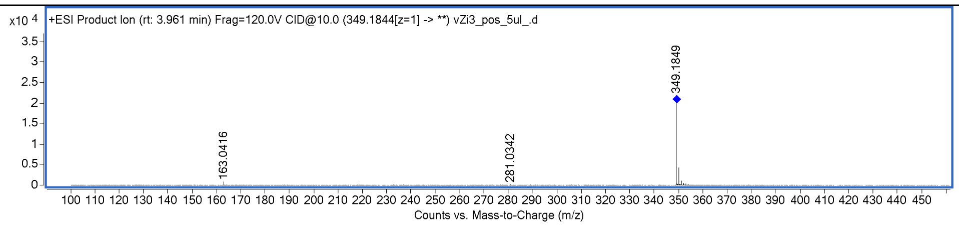
</td></tr>
</table>

Table S2. The relative compositions of the Z1–Z3 extracts. For the each studied compound (**1**–**26**), the HPLC peak areas representing the absolute amounts of a given compound within Z1, Z2 and Z3 extracts were rescaled, in order to sum to 1 in every row of the table below, expressing the relative compositions of the Z1–Z3 extracts.

		**Absolute Compositions**			**Relative Compositions (C_{rel})**		
		**Z1**	**Z2**	**Z3**	**Z1**	**Z2**	**Z3**
**1**	Chlorogenic acid	1540451	616767	1428191	0.4296	0.1720	0.3983
**2**	Neochlorogenic acid	3809788	1204722	4321964	0.4081	0.1290	0.4629
**4**	(*Z*)-Chlorogenic acid	7447554	1204722	4720773	0.5569	0.0901	0.3530
**6**	Caffeic acid	6243017	5526195	5075319	0.3706	0.3281	0.3013
**9**	Ziziphoroside isomer 1	559519	362891	241409	0.4808	0.3118	0.2074
**10**	Ziziphoroside isomer 2	175140	0	24764	0.8761	0.0000	0.1239
**11**	Ziziphoroside isomer 3	344946	251998	320946	0.3758	0.2745	0.3497
**13**	Rosmarinic acid	32458496	23080201	13968651	0.4670	0.3321	0.2010
**15**	Diosmin	12139796	21723047	12863281	0.2598	0.4649	0.2753
**23**	Acacetin	1838989	1331696	5276971	0.2177	0.1576	0.6247
**26**	Ursolic acid	11308	1391069	3039741	0.0025	0.3132	0.6843

Table S3. The relative biological activities of the Z1–Z3 extracts. Biological activities represented by 'survivability' of cells/enzymes (S) were taken from Figure 6 for the Z1–Z3 extracts of concentrations equal to 200 ug/mL and used to calculate biological activity (A) using formula A = 100% − S. For each biological activity test (I–VIII), the values of A for the Z1, Z2 and Z3 extracts were rescaled in order to sum to 1 in every row of the table below.

		**A = 100%-S [%]**			**Relative Activities (A_{rel})**		
		**Z1**	**Z2**	**Z3**	**Z1**	**Z2**	**Z3**
**I**	Cytotoxicity B16F10-10%	2.02	4.42	15.31	0.0929	0.2032	0.7039

**II**	Cytotoxicity B16F10-1%	2.22	6.4	16.95	0.0868	0.2503	0.6629
**III**	Cytotoxicity HaCat-10%	0	26.2	0	0.0000	1.0000	0.0000
**IV**	A375-10%	19.31	28.07	0	0.4076	0.5924	0.0000
**V**	SKMEL-3	8.14	23.12	0	0.2604	0.7396	0.0000
**VI**	MurT	29.93	49.75	17.4	0.3083	0.5125	0.1792
**VII**	MushT	7.6	9.07	0	0.4559	0.5441	0.0000
**VIII**	SOD	7.41	36.14	24.88	0.1083	0.5281	0.3636
